# *Gdf15* expression in thermogenic adipocytes regulates diet-induced weight gain in a sex-dependent manner

**DOI:** 10.1186/s10020-026-01505-5

**Published:** 2026-05-18

**Authors:** Jayashree Jena, Ayushi Sood, Joshua Peterson, Luis Miguel García-Peña, Eric T. Weatherford, Pritesh Patel, Shelly Roitershtein, David A. Smith, Vamsi Challa, Randy J. Seeley, Renata O. Pereira

**Affiliations:** 1 Department of Internal Medicine, Division of Endocrinology and Metabolism, Fraternal Order of Eagles Diabetes Research Center, University of Iowa, 169 Newton Road, 4322 PBDB, Iowa City, IA 52242 USA; 2 Department of Surgery, University of Michigan, Ann Arbor, MI USA

**Keywords:** GDF15, Obesity, Brown adipose tissue, Sex dimorphism

## Abstract

**Background:**

Growth differentiation factor 15 (GDF15) is a stress-induced cytokine known to bind to its endogenous receptor GDNF-family α-like (GFRAL) in the hindbrain, thereby modulating energy homeostasis. In response to prolonged high-fat diet (HFD) feeding, *Gdf15* expression is induced in various tissues, including liver and brown adipose tissue (BAT), leading to increased GDF15 serum levels. Although the liver is the primary source of circulating GDF15 during diet-induced obesity (DIO), other tissues are also required. We investigated whether BAT contributes to GDF15 circulating levels and if GDF15 induction in BAT regulates systemic metabolism during DIO.

**Methods:**

We generated mice with selective *Gdf15* deletion in thermogenic adipocytes (KO) and subjected them to 12 weeks of HFD feeding to determine the role of BAT-derived GDF15 on systemic metabolic homeostasis in both male and female mice.

**Results:**

Unexpectedely, despite no changes in GDF15 serum levels in mice fed *ad-libitum* regardless of sex or genotype, female KO mice were resistant to DIO, had increased energy expenditure and improved mitochondrial fatty acid oxidation in BAT, which was prevented by ovariectomy. Conversely, male KO mice had increased body weight and adiposity upon HFD feeding, along with reduced respiratory capacity in BAT mitchondria, and increased markers of fibro-inflammation.

**Conclusions:**

Together, our data reveal GDF15 induction in BAT is required to regulate weight gain in mice in a sex-dependent manner. Our results also suggest female sex hormones contribute to increase energy expenditure in female KO mice promoting leanness. Our study underscore the importance of rigorously addressing sex differences in GDF15 biology and pharmacology and suggests GDF15 might exert effects on energy balance and adiposity that are independent of signaling through GFRAL.

**Supplementary Information:**

The online version contains supplementary material available at 10.1186/s10020-026-01505-5.

## Introduction

Obesity and its associated comorbidities, such as type 2 diabetes mellitus (T2DM) and cardiovascular diseases (CVD), represent major public health issue in the United States and globally (Lyall et al. 2017; Fabbrini et al. 2010). Accumulating evidence indicates that brown adipose tissue (BAT) plays a central role in systemic metabloic homeostasis in rodents and in humans (Carpentier et al. 2023; Cypess et al. 2025). Recent studies in human subjects demonstrate a strong association between increased BAT activity with reduced prevalence of cardiometabolic diseases and healthier metabolic phenotypes in obese subjects (Becher et al. 2021; Herz et al. 2021). BAT activation not only increases thermogenesis but can also lead to secretion of signaling molecules, including peptides or batokines, that may act in autocrine, paracrine, and endocrine manners to improve systemic metabolic homeostasis (Cypess et al. 2025; Yang and Stanford 2022).

Growth differentiation factor 15 (GDF15) is a distant member of the transforming growth factor-β (TGFβ) superfamily that circulates as a ~ 25 kDa homodimer. Pre-clinical studies administering recombinant GDF15 in rodents and non-human primates revealed that GDF15 promotes anorectic effects and increases energy expenditure (Mullican et al. 2017; Wang et al. 2023; Benichou et al. 2023; Breit et al. 2023), thereby promoting weight loss via its central receptor, the glial-derived neurotrophic factor receptor α family-like specific receptor (GFRAL) (Baek and Eling 2019; Yang et al. 2017; Breit et al. 2021). Moreover, GDF15 serum levels are elevated in humans and rodents with obesity (Patel et al. 2019), and recent studies suggest that liver is the primary organ contributing to this rise in male mice (Xie et al. 2022; Patel et al. 2022). Importantly, we recently showed that, in the context of mitochondrial stress, GDF15 can be secreted as a batokine, contributing to resistance to diet-induced obesity (DIO) and glucose intolerance (Pereira et al. 2021; Jena et al. 2023). However, whether BAT contriubutes to GDF15 circulating levels during DIO, mediating changes in systemic metabolic homeostasis accross sexes had not been tested.

We generated mice selectively lacking GDF15 in thermogenic adipocytes (GDF15 BKO) to test the hypothesis that BAT-derived GDF15 is required to regulate metabolic homeostasis during DIO in mice. Our data indicate that *Gdf15* expression in thermogenic adipocytes is required to regulate diet-induced weight gain and adiposity in mice in a sex-dimorphic manner. While GDF15 BKO male mice are susceptible, female mice are resistant to DIO via mechanisms dependent on sex hormones. Importantly, these metabolic changes occur independently of alterations in GDF15 serum levels, suggesting GDF15-mediated local effects in BAT likely contribute to maintain energy balance in obesogenic coditions. Together, our studies highlight the importance of rigorously addressing sex differences in GDF15 biology and pharmachology, and warrants further investigation into possible GFRAL-independent effects driving GDF15-mediated changes in body weight regulation.

## Research design and methods

### Mouse model

Experiments were performed in male and female mice on the C57BL6/J background. *Gdf15*^fl/fl^ mice were generated as previously described (Jena et al. 2023). To generate mice lacking *Gdf15* selectively in thermogenic adipocytes (GDF15 BKO), *Gdf15*^fl/fl^ mice were crossed with transgenic mice expressing Cre recombinase under the control of the *Ucp1* promoter (Tg (Ucp1-cre)1Evdr), acquired from the Jackson Laboratories (#024670). WT controls harbored the *Gdf15* floxed alleles in homozygosis but lacked the *Ucp1* Cre. Mice were weaned at 3 weeks of age and were kept on standard chow (2920X Harlan Teklad, Indianapolis, IN). For DIO studies, six-week-old mice were fed HFD (60% kcal from fat, Research Diets, New Brunswick, NJ, USA, D12492) for 12 weeks. After 11 weeks of HFD-feeding, mice were singly housed in the Promethion System (Sable Systems International, Las Vegas, NV) at 30 °C to measure changes in resting metabolic rates, food intake, and locomotor activity. Unless otherwise specified, animals were housed at 22 °C with a 12-h light/dark cycle, free access to water, and standard chow or special diets. All animal experiments included in this study followed NIH animal research protocols and were approved by the University of Iowa IACUC (protocol #3032294).

### Ovariectomy surgeries

To determine the effects of sex hormones on the phenotype observed in female GDF15 BKO mice, ovariectomy surgeries were performed in 4- to 5-week-old WT and GDF15 BKO female mice. Additional details about the ovariectomy surgeries can be found in the supplementary file.

### Glucose and insulin tolerance tests, nuclear magnetic resonance, and serum analysis

Glucose tolerance tests (GTT) were performed after a 4-h fast for baseline studies and 6-h fast for HFD studies, and mice were administered glucose intraperitoneally (1 g/kg body weight), as described (Pereira et al. 2021). Insulin tolerance tests (ITT) were performed after a 2-h fast by injecting insulin intraperitoneally (0.75 U/kg body weight; Humulin, Eli Lilly, Indianapolis, IN). Plasma insulin was measured after 6-h fast using a commercially available kit according to the manufacturer’s directions (Ultra-Sensitive Mouse Insulin ELISA Kit, Chrystal Chem, Downers Grove, IL). Serum GDF15 (R&D Systems, Minneapolis, MN) was measured using kits available commercially according to the manufacturer’s directions. Whole-body composition was measured by nuclear magnetic resonance in the Bruker Minispec NF‐50 instrument (Bruker, Billerica, MA).

### Analysis of triglyceride levels

Hepatic triglyceride levels were measured using the EnzyChrom Triglyceride Assay Kit according to the manufacturer’s directions (BioAssay Systems, Hayward, CA), as previously described (Pereira et al. 2021).

### RNA extraction and quantitative RT-PCR

Total RNA was extracted from tissues using TRIzol reagent (Invitrogen, Waltham, MA) and purified with the RNeasy kit (QIAGEN Inc, Germantown, MD), as previously described (Pereira et al. 2021). Details about RT-PCR conditions and primer sequences can be found in the supplementary file.

### RNA Sequencing in BAT

Bulk RNA sequencing was performed in BAT of GDF15 BKO mice fed HFD for 12 weeks by the Iowa Institute of Human Genetics, Genomics Division, at the University of Iowa. Samples were quantified using the Trinean DropSense 16 (Pleasanton, CA) and quality assessed with the Agilent BioAnalyzer at the Iowa Institute of Human Genetics, Genomics Core. RNA-seq data have been deposited to the GEO database (accession number GSE296580). Additional details about RNA sequencing can be found in the supplementary file.

### Metabolomics

Whole BAT tissue was disrupted using bead mill homogenization in 18:1 (µl:mg wet tissue weight) ice-cold 2:2:1 methanol/acetonitrile/water extraction buffer and derivatized samples were analyzed by GC–MS. Heatmaps and enrichment analyses were performed using MetaboAnalyst 5.0. Details about sample prepration and GC–MS analysis are detailed in the supplementary file.

### Mitochondrial isolation and respirometry

Mitochondrial fraction was isolated from BAT and mitochondrial oxygen consumption rates were assessed using the Oroboros O_2_K Oxygraph system (Oroboros Instruments, Innsbruck, Austria), as previously described (Pereira et al. 2021; Jena et al. 2023). Details about mitochondrial isolation and functional assessment can be found in the supplementary file.

### Histology

Fragments of BAT were embedded in paraffin, portioned into 5‐μm‐thick sections, and stained with hematoxylin–eosin (Fisher, Pittsburgh, PA). Light microscopy was performed using a Nikon Eclipse Ti-S microscope (Nikon, Melville, NY).

### Western blot analysis

Immunoblotting analysis was carried out in BAT and iWAT, as previously described (Pereira et al. 2021). Protein extraction, immunoblot assay, and antibodies used in the present study are detailed in the supplementary file.

### Data analysis

All data are reported as mean ± SEM, unless otherwise noted. To establish statistical differences, Student’s t-test was performed for comparison of two groups, and One-Way or Two-Way ANOVA along with Tukey’s multiple-comparison test was employed when more than three groups were compared. For datasets where “n” was between 3–4, we performed the non-parametric Mann–Whitney test. A p ≤ 0.05 probability value was considered significantly different. GraphPad Prism Software was used to perform statistical calculations. CalR software (Mina et al. 2018) was used to analyze indirect calorimetry data collected in the Promethion system, including ANCOVA analysis for the regression plots.

## Results

### GDF15 expression in BAT is regulated in response to high-fat diet feeding

**Fig. 1:**
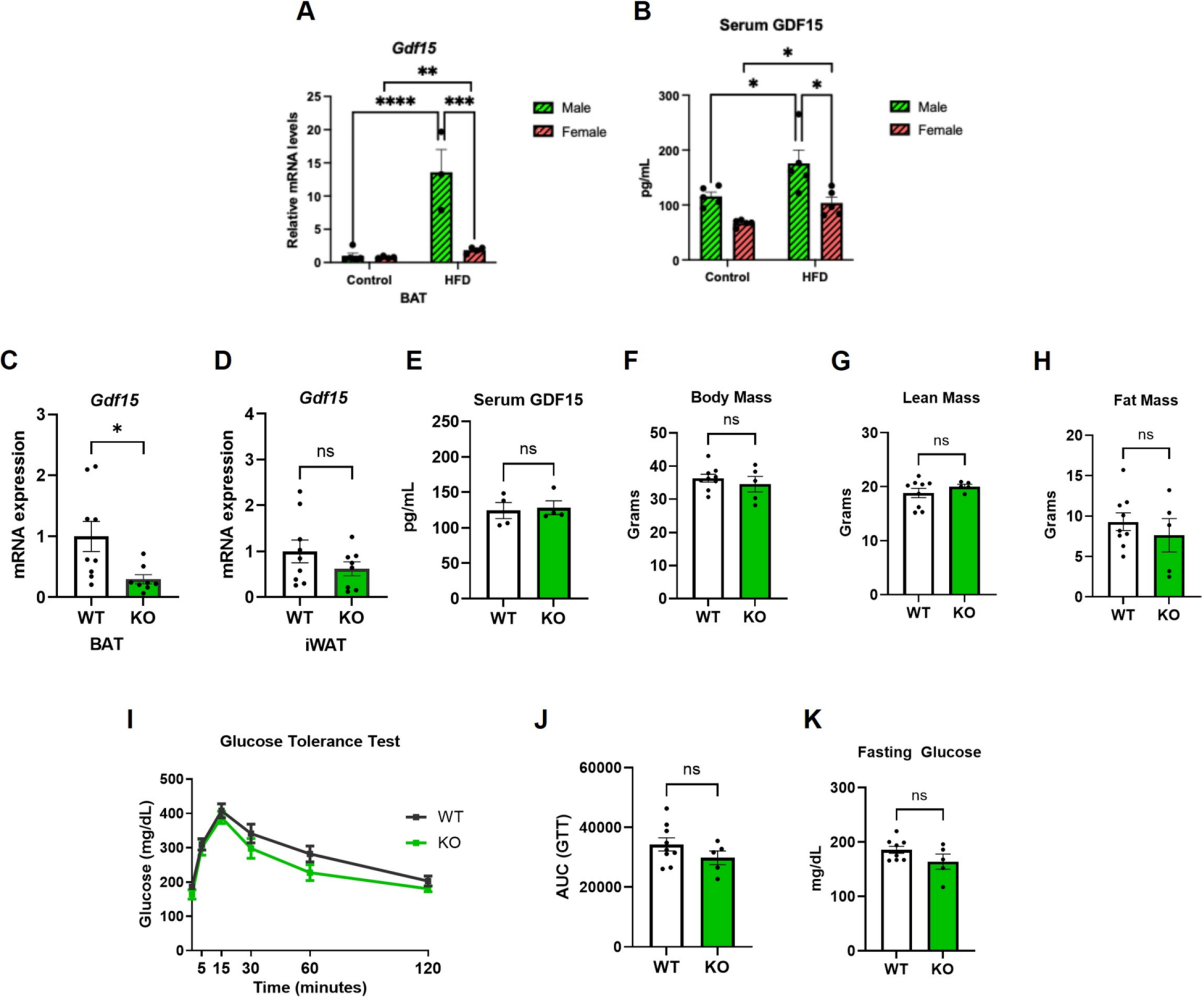
*Gdf15* expression in BAT is regulated in response to prolonged high-fat feeding. **A-B** GDF15 levels in 18-week-old male and female mice fed either control (10% fat content) or high-fat diet (HFD; 60% fat content) for 12 weeks. **A** Relative *Gdf15* mRNA levels in BAT of male (WT *n* = 6, KO *n* = 3) and female (WT *n* = 4, KO *n* = 4) mice normalized to *Tbp.* **B** Serum GDF15 levels in male (WT *n* = 5, KO *n* = 5) and female (WT *n* = 5, KO *n* = 5) mice. **C-K** Data collected under baseline conditions on 18-week-old in GDF15 BKO (KO) male mice and their WT littermate controls fed regular chow diet. **C** Relative *Gdf15* mRNA levels in BAT of male mice normalized to *Tbp* (WT *n* = 9, KO *n* = 8). **D** Relative *Gdf15* mRNA levels in iWAT of male mice normalized to *Tbp* (WT *n* = 9, KO *n* = 8). **E** Serum GDF15 in male mice (WT *n* = 5, KO *n* = 4). **F** Body mass. **G** Total lean mass. **H** Total fat mass. **I** Glucose tolerance test (GTT). **J** Area under the curve quantification for the GTT. **K** Fasting glucose level (WT *n* = 9, KO *n* = 5). Data are expressed as means ± SEM. Significant differences were determined by Two-Way ANOVA, Student’s *t*‐test or Mann–Whitney test, using a significance level of *p* ≤ 0.05. *p ≤ 0.05; **p ≤ 0.01; ***p ≤ 0.001; ****p ≤ 0.0001

GDF15 levels have been shown to increase in BAT after prolonged HFD feeding in male mice (Patel et al. 2019). First, we sought to confirm *Gdf15* upregulation in BAT in our model of DIO in both male and female mice. We measured *Gdf15* mRNA levels in WT mice fed either a control diet (10% calories from fat) or a HFD (60% calories from fat) for 12 weeks. *Gdf15* mRNA levels were significantly increased in BAT of both male and female mice (Fig. 1A). Similarly, GDF15 serum levels were also increased in male and female mice (Fig. 1B). Interestingly, although DIO induced GDF15 levels in both sexes, the response was attenuated in female mice (Fig. 1A and B).

To test the hypothesis that GDF15 induction in BAT during DIO contributes to regulate weight gain and metabolic homeostasis, we generated mice lacking the *Gdf15* gene conditionally in *Ucp1*-expressing/thermogenic adipocytes (GDF15 BKO mice). *Gdf15* mRNA (Fig. 1C) and protein (Supplemetary Fig. 1A and B) levels were significantly reduced in BAT of male GDF15 BKO mice, but were unchanged in other tissues, including inguinal white adipose tissue (iWAT; Fig. 1D). Of note, reduced *Gdf15* mRNA levels in BAT of GDF15 BKO mice are due to downregulation specifically in mature brown adipocytes (Supplementary Fig. 1 C) and not in the stromal vascular fraction (SVF) (Supplementary Fig. 1 B) in mice fed regular chow. GDF15 serum levels were unchanged between WT littermate control mice and GDF15 BKO mice (Fig. 1E).

### Body composition and glucose homeostasis are unchanged in GDF15 BKO male mice fed regular chow

We previously demonstrated that *Gdf15* deletion in BAT did not affect body weight, body composition, or glucose homeostasis in 6-week-old male GDF15 BKO mice fed regular chow (Jena et al. 2023). Here, we confirmed these results in 6- and 20-week-old male mice. Body mass (Fig. 1F and Supplementary Fig. 1E), total lean mass (Fig. 1G and Supplementary Fig. 1 F), and total fat mass (Fig. 1H and Supplementary Fig. 1G) were unchanged between genotypes, regardless of age. Likewise, glucose homeostasis, as measured by glucose tolerance tests (GTT; Fig. 1I and J and Supplementary Fig. 1H and I) and fasting glucose levels (Fig. 1K and Supplementary Fig. 1 J and K), were unaffected by *Gdf15* deletion in thermogenic adipocytes irrespective of age.

### GDF15 BKO male mice are susceptible to DIO

**Fig. 2:**
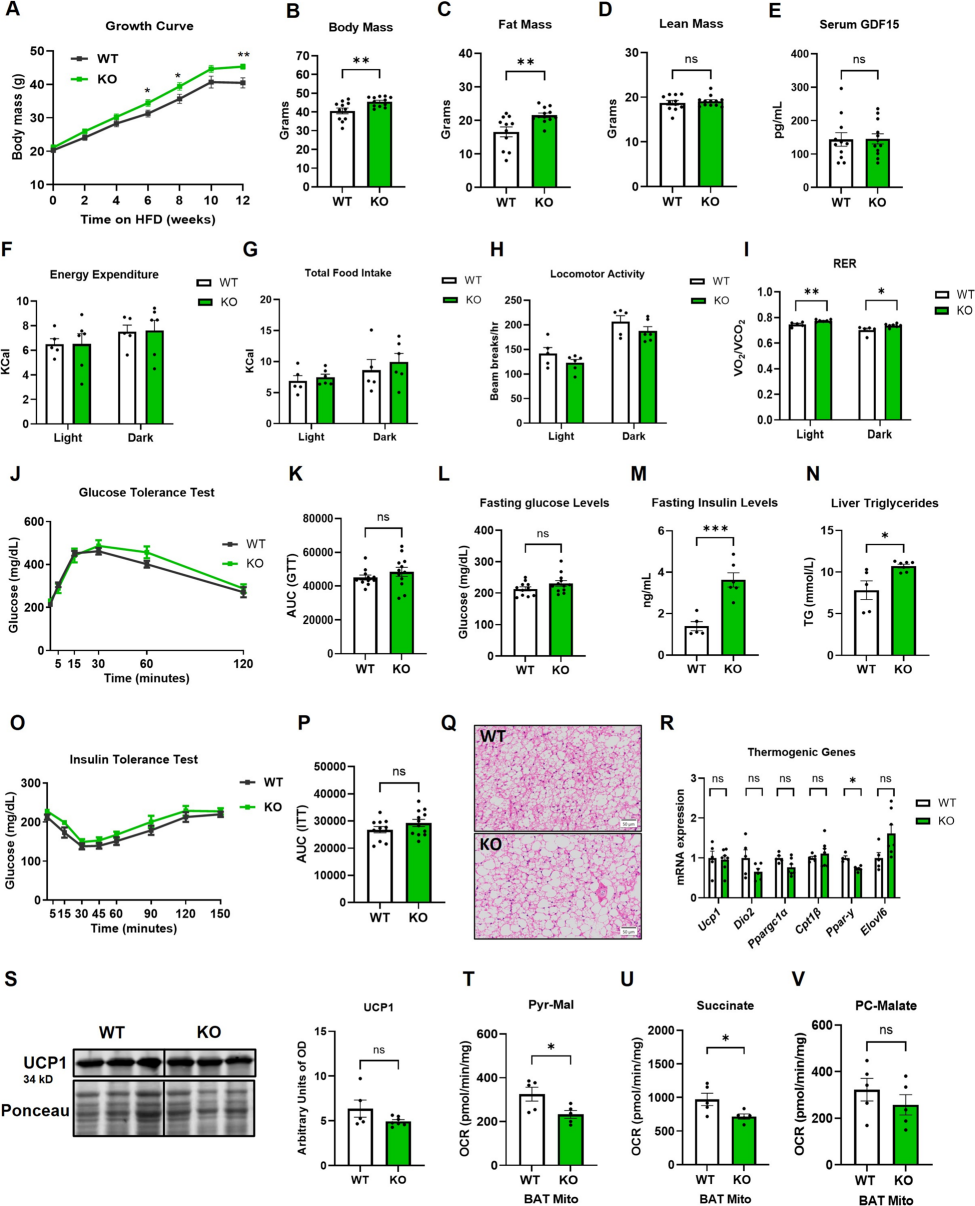
GDF15 BKO male mice are susceptible to diet induced obesity (DIO) and insulin resistance. Data was collected in 18-week-old male GDF15 BKO (KO) and WT littermate controls fed high-fat diet for 12 weeks. **A** Body weight curve during DIO protocol. **B** Body mass. **C** Total fat mass. **D** Total lean mass (WT *n* = 11, KO *n* = 12). **E** GDF15 serum levels. **F**-**I** Indirect calorimetry data represented as the average for light and dark cycles during the last 48 h of data recording. **F** Energy expenditure. **G** Total food intake. **H** Locomotor activity. **I** Respiratory exchange ratio (RER) (WT n = 5, KO *n* = 5). **J** Glucose tolerance test (GTT). **K** Area under the curve quantification for the GTT. **L** Fasting glucose levels (WT *n* = 11, KO *n* = 12). **M** Fasting insulin levels (WT *n* = 5, KO *n* = 7). **N** Liver triglycerides levels (WT *n* = 5, KO *n* = 7). **O** Insulin tolerance test (ITT). **P** Area under the curve quantification for the ITT (WT *n* = 11, KO *n* = 12). **Q** Representative images of hematoxylin and eosine-stained histological sections of BAT (WT *n* = 3, KO *n* = 3). Scale bar = 50 µm. **R** Relative mRNA expression of thermogenic genes in BAT normalized to *Tbp* (WT *n* = 5, KO *n* = 7). **S** Representative immunoblots and respective densitometric quantification for UCP1 in BAT normalized to Ponceau (WT *n* = 5, KO *n* = 7). OD: optical density. (T-V) Oxygen consumption rates in mitochondria isolated from BAT. **T** Pyruvate/malate (Pyr-Mal)-supported state 2 respirations. **U** Succinate-supported state 2 respirations. **V** Palmitoyl-carnitine (PC)/malate-supported state 2 respirations (WT *n* = 5, KO *n* = 5). Data are expressed as means ± SEM. Significant differences were determined by Student’s *t*‐test, using a significance level of *p* ≤ 0.05. *P ≤ 0.05; **p ≤ 0.01

*Gdf15* global knockout mice have been reported to have an exacerbated response to DIO, at least in part, due to increases in food intake (Patel et al. 2022; Tran et al. 2018). Here, we hypothesized that BAT-derived GDF15 contributes to GDF15 circulating levels and is required to regulate energy homeostasis during DIO. To test this hypothesis, we fed 6-week-old male GDF15 BKO mice HFD for a total of 12 weeks. Male KO mice gained significantly more weight relative to their WT littermate controls as shown in the weekly body weight curve (Fig. 2A) and their final body weights (Fig. 2B). Total fat mass (Fig. 2C) was significantly higher in KO male mice relative to their WT controls, while total lean mass was unchanged between genotypes (Fig. 2D). Noteworthy, although *Gdf15* mRNA levels were significantly reduced in BAT of KO mice (Supplementary Fig. 2 A), GDF15 serum levels in mice with *ad libitum* access to HFD (Fig. 2E) or after a 4-h fast (Supplementary Fig. 2B) were unchanged between genotypes. *Gdf15* mRNA levels were unchanged in iWAT (Supplementary Fig. 2 C), but were significantly increased in the liver of male KO mice relative to WT mice (Supplementary Fig. 2D), likely contributing to maintain normal GDF15 serum levels and compensating for lack of GDF15 in BAT.

To gain insight into the mechanisms contributing to these changes in weight gain, we placed a subset of WT and KO male mice fed HFD in metabolic chambers. Energy expenditure (Fig. 2F and Supplementary Fig. 2E), total food intake (Fig. 2G), and locomotor activity (Fig. 2H) were unchanged between genotypes, while respiratory exchange ratio (RER) was significantly increased in KO males during both the light and dark cycles (Fig. 2I). While KO males have increased body weight relative to WT mice, glucose homeostasis, as demonstrated by GTT (Fig. 2J and K) and fasting glucose levels (Fig. 2L) were similar between genotypes. Fasting insulin levels (Fig. 2M) and liver triglyceride levels were significantly elevated in KO mice, indicating exacerbated hepatic steatosis (Fig. 2N). Surprisingly, despite changes in fasting insulin levels, we detected no significant differences in insulin tolerance tests (ITT) between genotypes (Fig. 2O and P), indicating insulin sensitivity is largely preserved.

Morphologically, brown adipocytes in male KO mice had enlarged unilocular lipid droplets, suggesting exacerbated lipid accumulation relative to WT mice (Fig. 2Q), however, most thermogenic genes in BAT were unchanged between genotypes, except for a modest reduction in *Pparγ* levels (Fig. 2R). Likewise, we detected no changes in the expression of various fatty acid oxidation (FAO) genes (Supplementary Fig. 2 F) or in other genes involved in lipid metabolism (Supplementary Fig. 2G), except for a small reduction in *Gpat* mRNA levels in BAT. Moreover, UCP1 protein levels (Fig. 2S) as well as protein levels of enzymes involved in lipolysis and de novo lipogenesis (Supplementary Fig. 2H and I) were unchanged between WT and male KO mice.

Mitochondria play a critical role in regulating BAT function, thereby contributing to whole body metabolism (Pereira et al. 2021; Wikstrom et al. 2014; Pisani et al. 2018). Therefore, to assess mitochondrial function, we performed high resolution respirometry in mitochondria isolated from BAT. Mitochondria oxygen consumption rates were significantly reduced in pyruvate/malate (Fig. 2T) and succinate-supported state 2 respirations (Fig. 2U) in KO male mice, whereas palmitoyl-carnitine/malate-supported respirations were not significantly changed between genotypes (Fig. 2V). Nonetheless, immunoblotting for oxidative phosphorylation (OXPHOS) complexes showed similar levels of complexes I, II, III and V in whole BAT protein lysates between WT and KO male mice (Supplementary Fig. 2 J and K), suggesting no reduction in overall mitochondrial mass between genotypes.

Metabolic changes in BAT may be accompanied by induction of iWAT browning in mice (Pereira et al. 2021; Wikstrom et al. 2014; Pisani et al. 2018), however, markers of browning, such as UCP1 and PGC-1α protein levels were unchanged in iWAT of KO mice relative to their WT littermate controls (Supplementary Fig. 2L and M). Although *Ucp1* levels were transcriptionally induced, *Ppargc1α* and *Cpt1β* were reduced in iWAT of KO mice (Supplementary Fig. 2 N). Finally, expression of FAO genes was unchanged between genotypes in iWAT (Supplementary Fig. 2O), indicating that iWAT browning is likely unaffected in male mice when *Gdf15* is deleted in BAT. Together, our data suggest that *Gdf15* deletion in BAT of male mice results in increased weight gain and adiposity, and reduced mitochondrial respiratory capacity without major changes in lipid metabolic pathways or in GDF15 circulating levels.

### Global transcriptomic analysis in BAT reveals increased markers of fibro-inflammation in GDF15 BKO male mice fed HFD

**Fig. 3:**
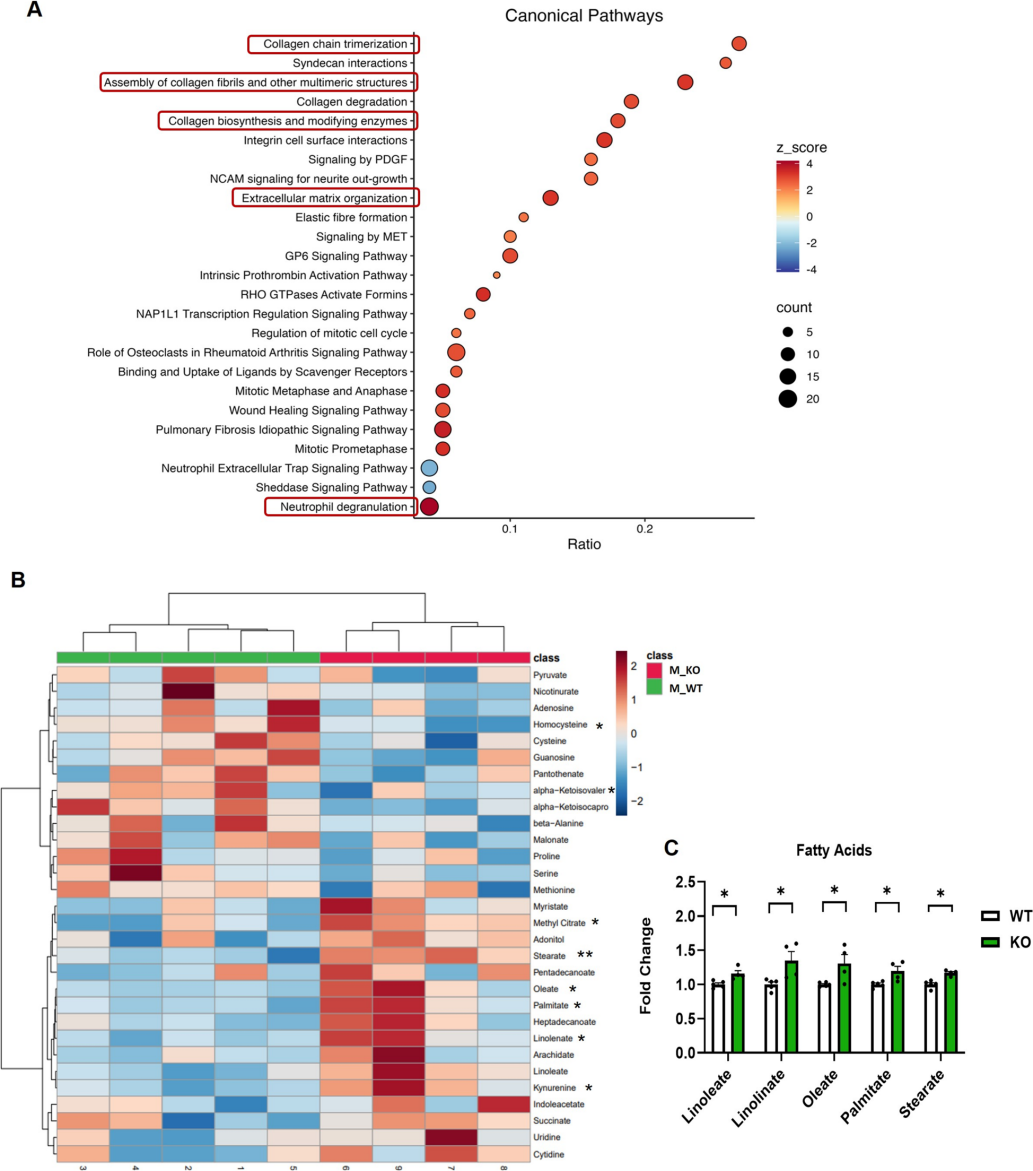
Global transcriptomic and metabolomic analysis in BAT of GDF15 BKO male mice fed HFD. Data was collected in 18-week-old male GDF15 BKO (KO) and WT littermate controls fed high-fat diet for 12 weeks. **A** Bubble plot showing the top 25 canonical pathways from the Ingenuity Pathway Analysis (IPA) database containing genes with a significant overlap (adjusted *p*-value ≤ 0.05) to those differentially expressed in GDF15 BKO mice. Size of the bubble indicates the -log of the adjusted *p*-value (Benjamini–Hochberg) from the pathway analysis. Plotted on the x-axis is the ratio of differentially expressed genes relative to the number of genes in the pathway. Bubble color indicates z-score, which indicates the predicted activation (positive) or inhibition (negative) of the pathway based on the directionality of gene changes GDF15 BKO relative to WT mice. Noteworthy, pathways predicted to be upregulated are highlighted in red. **B** Heatmap of the top 30 differentially enriched metabolites in male KO BAT (WT *n* = 5, KO *n* = 4). **C** Bar graph depicting fold change of various fatty acids in GDF15 BKO male versus WT mice (WT *n* = 5, KO *n* = 4). Data are expressed as means ± SEM. Significant differences were determined by Student’s *t*‐test or Mann–Whitney test, using a significance level of *p* ≤ 0.05. *P ≤ 0.05; **p ≤ 0.01

To gain further insight into local molecular changes potentially contributing to BAT dysfunction in male GDF15 BKO mice, we performed bulk RNA sequencing (RNASeq) in BAT collected from WT and GDF15 BKO mice after 12 weeks of high-fat feeding. Ingenuity Pathway Analysis revealed that pathways involved in collagen biosynthesis, fibrosis, and extracellular matrix (ECM) remodeling were amongst the top canonical pathways predicted to be induced in GDF15 deficient mice (Fig. 3A). Likewise, pathways related to neutrophil degranulation and inflammation were also upregulated in BAT of GDF15 BKO mice (Fig. 3A). Heatmap of the top 50 differentially expressed genes in WT vs. GDF15 BKO showed upregulation of genes encoding for various collagens (Supplementary Fig. 3 A). Genes involved in ECM remodeling, such as *Timp2* and *Fn1*, were also induced in BAT of KO mice relative to WT controls (Supplementary Fig. 3 A). Induction of *Col1a1*, *Col1a2,* and *Timp2* was confirmed by qPCR in BAT of GDF15 BKO mice (Supplementary Fig. 3B). Furthermore, *Ccl6*, a chemokine involved in macrophage recruitment and *Cd68*, a macrophage marker, were also upregulated in KO mice, suggesting increased recruitment of inflammatory cells to BAT (Supplementary Fig. 3 A). Additional macrophage markers, namely, *F4/80* and *Mcp1,* were found to be induced in BAT of KO male mice when measured by qPCR (Supplementary Fig. 3 C). Together, our data suggest that deletion of *Gdf15* in BAT exacerbates diet-induced fibro-inflammation in male mice.

### Metabolomic analysis shows increased accumulation of fatty acids in BAT of GDF15 BKO male mice fed HFD

To assess global metabolite changes, we conducted GC–MS analysis in BAT samples from WT and GDF15 BKO mice after 12 weeks of HFD feeding. Enrichment analysis of the top 25 metabolite sets identified in our study highlights changes in lipid metabolism processes, including fatty acid biosynthesis and beta oxidation (Supplementary Fig. 3D). Indeed, heatmap of the top 30 differentially enriched metabolites shows increased accumulation of various fatty acids in male KO BAT, including, oleate, stearate, and palmitate (Fig. 3B). This is also highlighted in Fig. 3C, depicting fold change versus WT mice for various fatty acids. Additional changes identified in our analysis include significant reductions in homocysteine and α-Ketoisovalerate (KIV), which are byproducts of amino acid metabolism, and may affect mitochondria and brown adipocyte function (Verkerke et al. 2024).

### Body composition and glucose homeostasis are unchanged in GDF15 BKO female mice fed regular chow diet

**Fig. 4:**
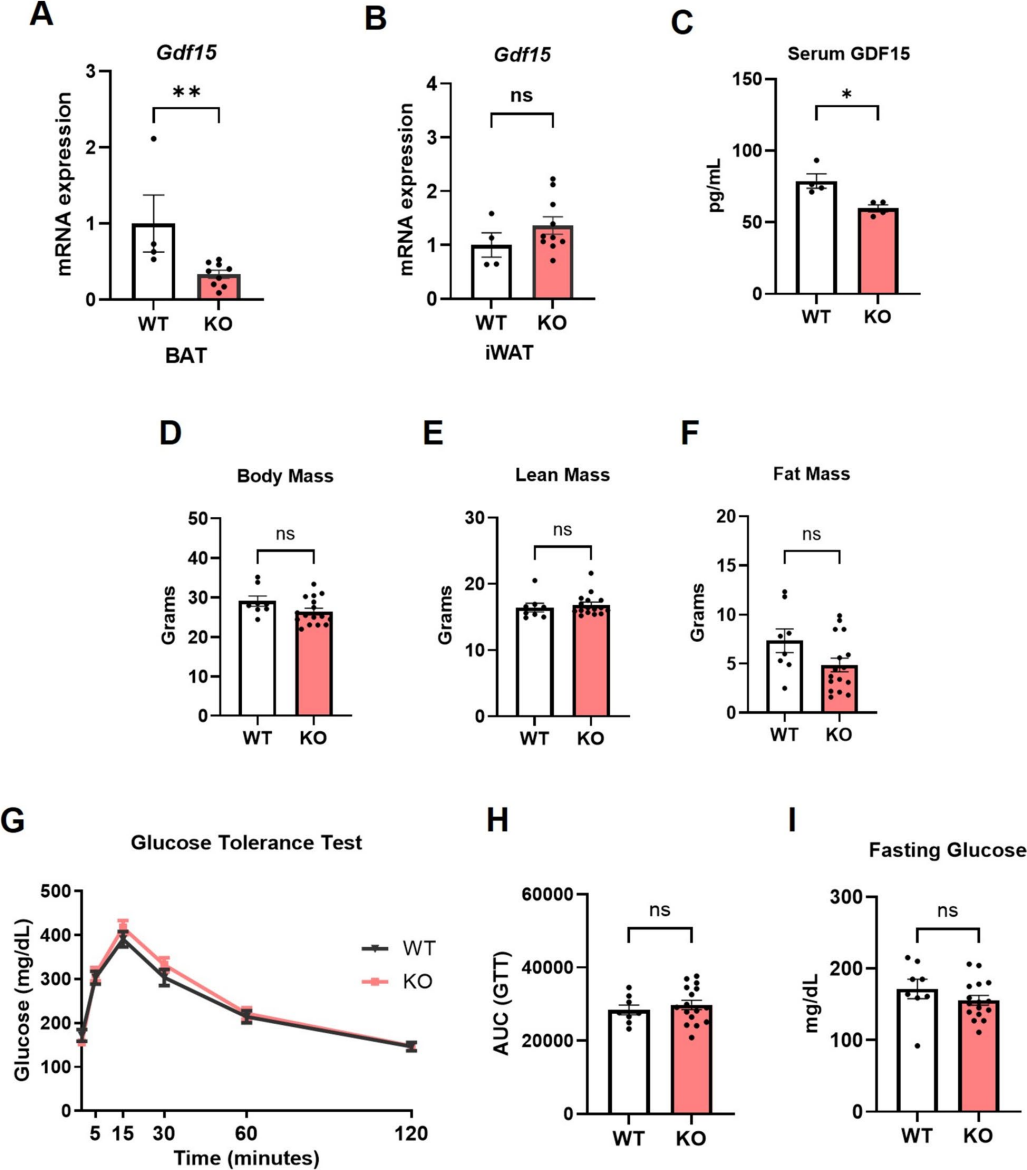
Body composition and glucose homeostasis in GDF15 BKO female mice fed regular chow diet. Data collected under baseline conditions on 18-week-old GDF15 BKO (KO) female mice and their WT littermate controls fed regular chow diet. **A** Relative *Gdf15* mRNA levels in BAT of female mice normalized to *Tbp* (WT *n* = 4, KO *n* = 9). **B** Relative *Gdf15* mRNA levels in iWAT of female mice normalized to *Tbp* (WT *n* = 4, KO *n* = 9). **C** Serum GDF15 in female mice (WT *n* = 4, KO *n* = 4). **D-I** Metabolic data in females (WT *n* = 8, KO *n* = 16). **D** Body mass. **E** Total lean mass. **F** Total fat mass. **G** Glucose tolerance test (GTT). **H** Area under the curve quantification for the GTT. **I** Fasting glucose level (WT *n* = 8, KO *n* = 16). Data are expressed as means ± SEM. Significant differences were determined by Student’s *t*‐test or Mann–Whitney test, using a significance level of *p* ≤ 0.05. *p ≤ 0.05

In parallel with our studies in male GDF15 BKO mice, we also performed studies in females. Female GDF15 BKO mice had significantly reduced *Gdf15* mRNA levels in BAT (Fig. 4A), but not in iWAT (Fig. 4B), brain (Supplementary Fig. 4 A), kidney (Supplementary Fig. 4B) or liver (Supplementary Fig. 4 C), confirming tissue specific deletion. Interestingly, GDF15 serum levels were slightly reduced in female GDF15 BKO mice relative to littermate controls (Fig. 4C). As shown in male mice, body weight (Fig. 4D and Supplementary Fig. 4D), total lean mass (Fig. 4E and Supplementary Fig. 4E) and total fat mass (Fig. 4F and Supplementary Fig. 4 F) were unchanged between genotypes irrespective of age in chow-fed female mice (6 or 20 weeks). Likewise, glucose homeostasis, as measured by GTT (Fig. 4G and H and Supplementary Fig. 4G and H) and fasting glucose levels (Fig. 4I and Supplementary Fig. 4I) were unaffected by *Gdf15* deletion in thermogenic adipocytes of female mice regardless of age.

### GDF15 BKO female mice are resistant to DIO

**Fig. 5:**
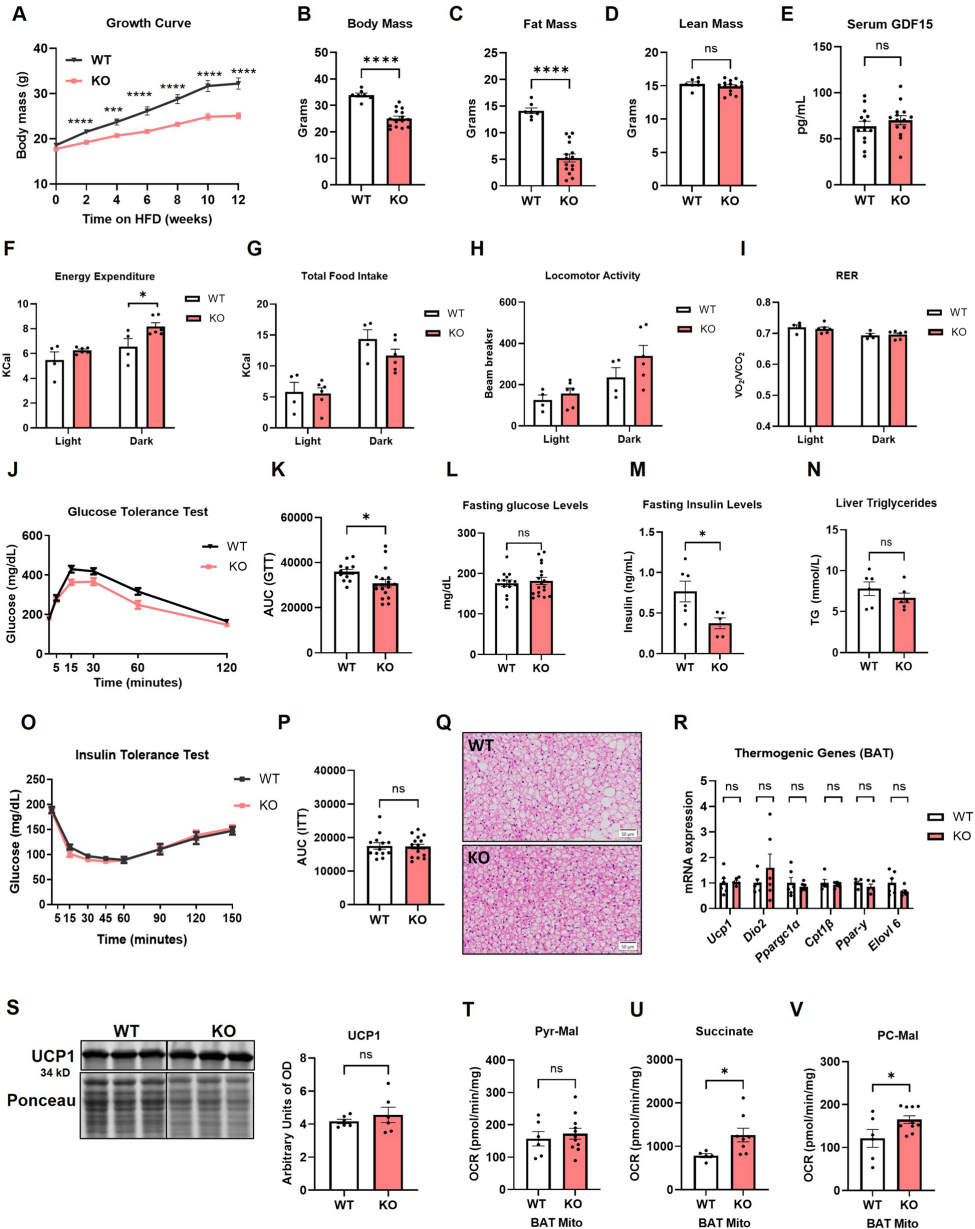
GDF15 BKO female mice are resistant to DIO and insulin resistance. Data was collected in 18-week-old female GDF15 BKO (KO) and WT littermate controls fed high-fat diet for 12 weeks. **A** Body weight curve during DIO protocol (WT *n* = 13, KO *n* = 15). **B** Body mass. **C** Total fat mass. **D** Total lean mass (WT *n* = 7, KO *n* = 13). **E** GDF15 serum levels (WT *n* = 13, KO n = 14). **F-I** Indirect calorimetry data represented as the average for light and dark cycles during last 48 h of data recording. **F** Energy expenditure. **G** Food intake. **H** Locomotor activity. **I** Respirartory exchange ratio (WT *n* = 4, KO *n* = 6). **J** Glucose tolerance test (GTT). **K** Area under the curve quantification for GTT. **L** Fasting glucose levels (WT *n* = 13, KO *n* = 18). **M** Fasting insulin levels (WT *n* = 6, KO *n* = 5). **N** Liver triglycerides levels (WT *n* = 6, KO *n* = 6). **O** Insulin tolerance test (ITT). **P** Area under the curve quantification for the ITT (WT *n* = 13, KO *n* = 17). **Q** Representative images of hematoxylin and eosine-stained histological sections of BAT (WT *n* = 3, KO *n* = 3). Scale bar = 50 µm. **R** Relative mRNA expression of thermogenic genes in BAT normalized to *Tbp* (WT *n* = 6, KO *n* = 6). **S** Representative immunoblots and quantification for UCP1 in BAT normalized to ponceau or beta-actin (B-actin) (WT *n* = 6, KO *n* = 6). OD-optical density. **T-V** Oxygen consumption rates in mitochondria isolated from BAT. **T** Pyruvate/malate (Pyr-Mal)-supported state 2 respirations. **U** Succinate-supported state 2 respirations. **V** Palmitoyl-carnitine (PC)/malate-supported state 2 respirations (WT *n* = 6, KO *n* = 11). Data are expressed as means ± SEM. Significant differences were determined by Student’s *t*‐test, using a significance level of *p* ≤ 0.05. *p ≤ 0.05; ***p ≤ 0.001; ****p ≤ 0.0001

Surprisingly, in contrast to male GDF15 BKO mice, female GDF15 BKO mice had reduced body weight relative to their WT controls. After two weeks of HFD feeding, we observed that KO female mice had lower body weight, which persisted throughout the DIO protocol (Fig. 5A). Final body mass (Fig. 5B) and total fat mass (Fig. 5C) were also significantly lower in KO female mice relative to their WT controls, while total lean mass was unchanged between genotypes (Fig. 5D). As observed in males, despite reduced *Gdf15* mRNA levels in BAT (Supplementary Fig. 5 A), GDF15 serum levels in *ad libitum*-fed mice were unchanged between genotypes after 12 weeks of HFD (Fig. 5E). Interestingly, unlike males, KO females had a significant reduction in GDF15 serum levels after a 4-h fast (Supplementary Fig. 5B). *Gdf15* mRNA levels were unchanged in iWAT (Supplementary Fig. 5 C) and in the liver (Supplementary Fig. 5D) of female KO mice relative to their WT controls. Reduced body weight was associated with higher energy expenditure during the dark cycle (Fig. 5F and Supplementary Fig. 5E). Conversely, food intake (Fig. 5G), locomor activity (Fig. 5H) and respiratory exchange ratio (Fig. 5I) remained unchanged between genotypes. Glucose homeostasis was slightly improved in GDF15 KO females, as shown by the lower area under the curve (AUC) for the GTT (Fig. 5 J and K), although fasting glucose levels were unchanged between genotypes (Fig. 5L). Fasting insulin levels were reduced in KO females (Fig. 5M), while liver triglyceride levels (Fig. 5N) and ITT (Fig. 5O and P) were similar between KO female mice and their WT littermate controls. Histologically, BAT morphology in KO females showed smaller adipocytes with multilocular lipid droplets, suggesting reduced lipid accumulation (Fig. 5Q) relative to WT mice. Molecularly, expression of thermogenic genes (Fig. 5R) and UCP1 protein levels (Fig. 5S) remained unchanged between genotypes. Furthermore, we found no differences in genes involved in FAO (Supplementary Fig. 5 F) or other lipid metabolism processes (Supplementary Fig. 5G). Protein levels of enzymes involved in lipolysis and de novo lipogenesis were also unchanged in BAT between WT and KO female mice (Supplementary Fig. 5H and I). Unlike males, mitochondrial oxygen consumption rates were unchanged in pyruvate/malate supported state 2 respirations (Fig. 5T), whereas succinate- (Fig. 5U) and palmitoyl-carnitine/malate-supported (Fig. 5V) respirations were significantly elevated in female KO mice. OXPHOS protein levels in BAT were unchanged between genotypes, except for complex III, which was slightly increased in female KO mice, relative to WT littermate controls (Supplementary Fig. 5 J and K).

To asses browning of iWAT, we measured UCP1 and PGC-1α protein levels (Supplementary Fig. 5L and M) and thermogenic gene expression (Supplementary Fig. 5 N), but did not detect any significant differences between genotypes. Similarly, mRNA levels of FAO genes (Supplementary Fig. 5O) were unchanged in iWAT between WT and KO female mice. Together, our data suggest *Gdf15* deletion in BAT attenuates DIO in female mice, likely by driving increases in energy expenditure.

### Genes encoding TCA cycle and pyruvate metabolism enzymes are inhibited, while genes supporting protein translation are induced in GDF15 BKO females

**Fig. 6:**
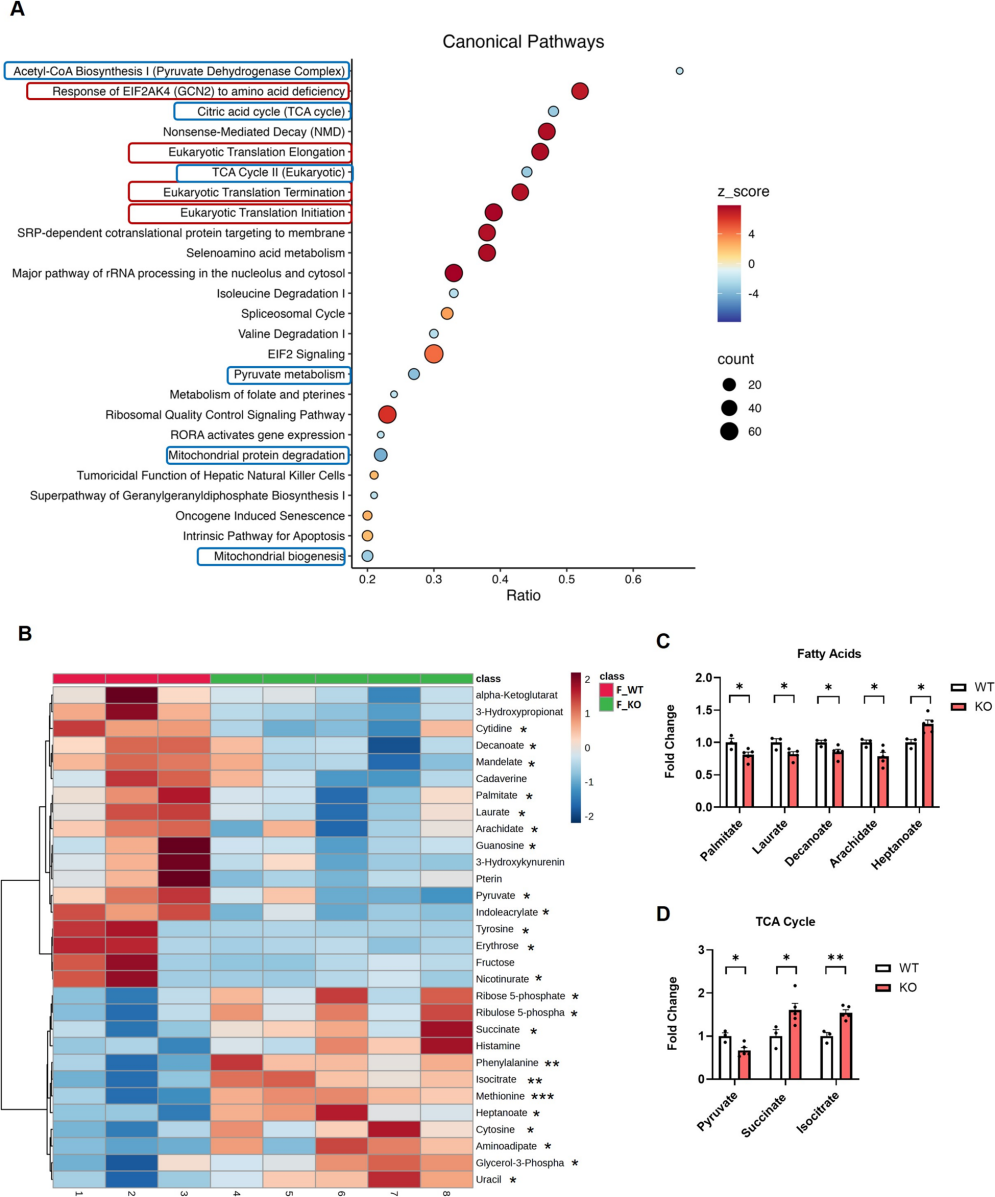
Global transcriptomic and metabolomic analysis in BAT of GDF15 BKO female mice fed HFD. Data was collected in 18-week-old female GDF15 BKO (KO) and WT littermate controls fed high-fat diet for 12 weeks. **A** Bubble plot showing top 25 canonical pathways from the Ingenuity Pathway Analysis (IPA) database containing genes with a significant overlap (adjusted *p*-value ≤ 0.05) to those differentially expressed in GDF15 BKO mice. Size of bubble indicates -log of adjusted *p*-value (Benjamini–Hochberg) from the pathway analysis. Plotted on the x-axis is the ratio of differentially expressed genes relative to the number of genes in the pathway. Bubble color indicates z-score, which indicates predicted activation (positive) or inhibition (negative) of pathway based on directionality of gene changes in GDF15 BKO relative to WT mice. Noteworthy pathways predicted to be upregulated are highlighted in red and those predicted to be downregulated are highlighted in blue. **B** Heatmap of top 30 differentially enriched metabolites in female KO BAT. **C** Bar graph depicting fold change of various fatty acids in GDF15 BKO female versus WT mice. **D** Bar graph depicting fold change of various TCA cycle metabolites in GDF15 BKO female versus WT mice (WT *n* = 3, KO *n* = 5). Data are expressed as means ± SEM. Significant differences were determined by Student’s *t*‐test, using a significance level of *p* ≤ 0.05. *p ≤ 0.05; **P ≤ 0.01; ***p ≤ 0.001

Canonical pathway analysis on RNASeq data in BAT of female GDF15 BKO versus WT littermate control mice following HFD feeding revealed an overall downregulation of genes encoding for proteins involved in TCA cycle, pyruvate metabolism, mitochondrial biogenesis and mitochondrial protein degradation (Fig. 6A). Conversely, genes encoding for ribosomal proteins, ribosomal quality control and involved in translation processes were upregulated (Fig. 6A). A heatmap depicting the top 50 differentially expressed genes in female KO vs. WT mice shows that genes involved in glucose metabolism and adipogenesis were down regulated in KO female mice, including *Pfkfb2*, *Gsk3b*, *Ppara* and *Sox5* (Supplementary Fig. 6 A). Importantly, the fibrosis (Supplementary Fig. 6B) and macrophage genes (Supplementary Fig. 6 C) induced in male mice were unchanged in KO females. Interestingly, although pathways related to protein translation were induced in KO female mice, we detected no changes in phosphorylation status of the eukaryotic translation initiation factor 2 A (eIF2A) between WT and KO mice (Supplementary Fig. 6D). Together, these data indicate that deletion of GDF15 in BAT of female mice fed HFD leads to transcriptional changes consistent with reduced pyruvate and glucose metabolism and increased ribosomal biogenesis, suggesting increased translational capacity.

### Metabolomic analysis suggests increased fatty acid utilization and altered TCA intermediates in BAT of GDF15 BKO female mice fed HFD

We conducted GC–MS analysis in BAT samples from WT and GDF15 BKO female mice after 12 weeks of HFD feeding to assess global metabolite changes. Enrichment analysis of the top 25 metabolite sets showed changes in fatty acid metabolism and beta oxidation as well as in the citric acid cycle (TCA; Supplementary Fig. 6E). Contrary to what we observed in male mice, heatmap of the top 30 differentially enriched metabolites in females showed reduced accumulation of various fatty acids, including palmitate, laurate, and arachidate (Fig. 6B and C), consistent with increased fatty acid utilization or decreased fatty acid synthesis in females. Indeed, respirometry in isolated BAT mitochondria indicated increased capacity for fatty acid oxidation in female mice (Fig. 5B). In contrast, the TCA cycle intermediates succinate and isocitrate were increased in females (Fig. 6D). This agrees with our RNASeq showing downregulation of TCA-related pathways. Together these findings suggest that GDF15 deletion in female mice results in downregulation of TCA cycle enzymes, while inducing a compensatory increase in fatty acid utilization, indicating a unique metabolic adaptations in BAT of females relative to males. Further studies assessing enzyme levels and activity will be required to confirm these metabolic changes, as the function of mitochondrial proteins can be heavily regulated posttranslationally (Yang et al. 2024; Stram and Payne 2016), which is not captured by transcriptomic and metabolomic data alone. Additional changes identified in our analysis included significant accumulation of methionine (Fig. 6B), a metabolite important for redox balance, polyamine synthesis and epigenetic regulation (Navik et al. 2021).

### Ovariectomized female GDF15 BKO mice lack the resistance to DIO

**Fig. 7:**
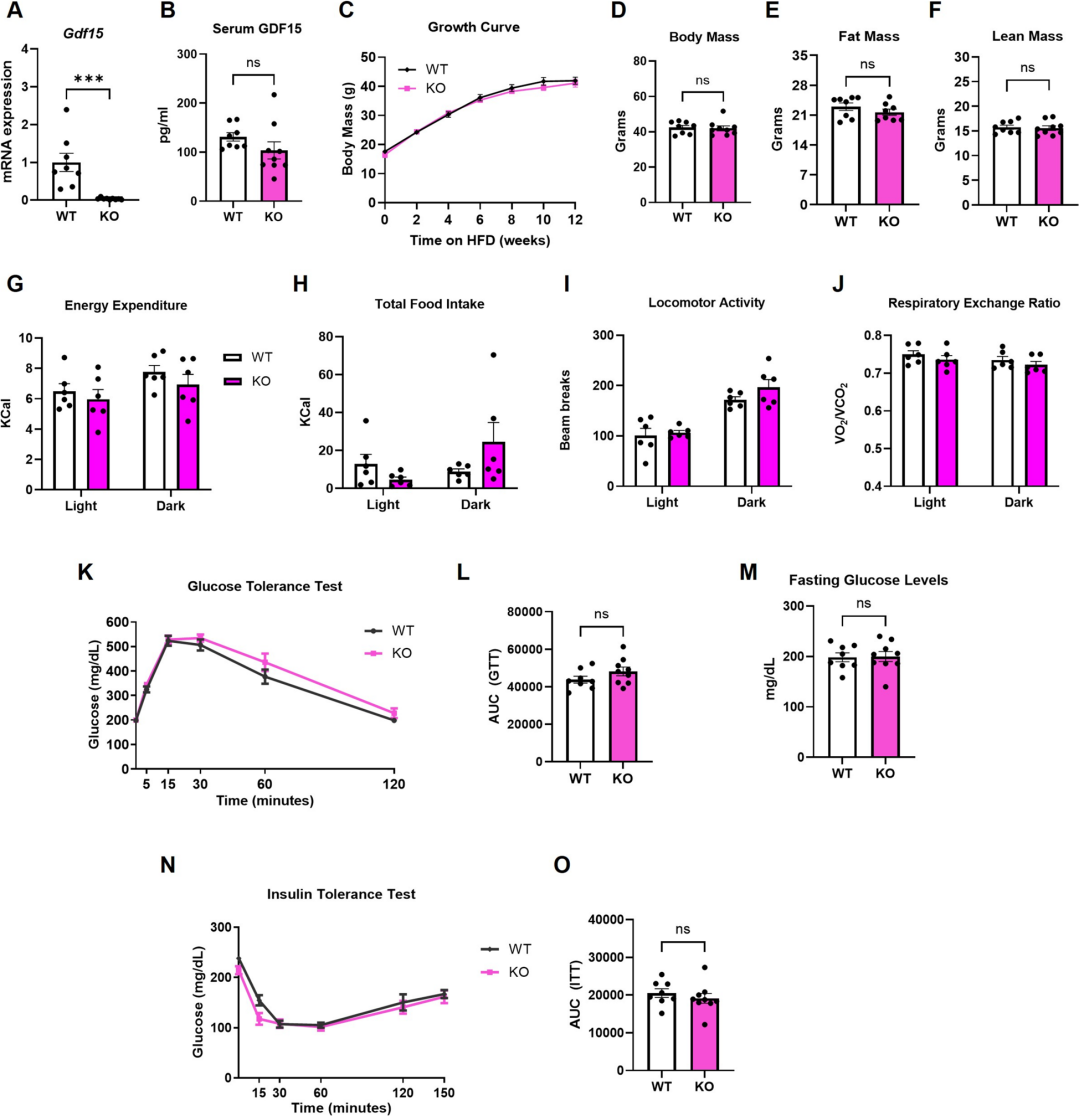
Ovariectomized GDF15 BKO mice lack resistance to DIO. Data collected in female WT or GDF15 BKO mice kept on HFD (60% fat content) for a period of 12 weeks after undergoing bilateral ovariectomy surgeries (OVX). **A** Relative *Gdf15* mRNA levels in BAT normalized to *Tbp* (WT *n* = 8, KO *n* = 9). **B** GDF15 serum levels. **C** Body weight curve during diet-induced obesity (DIO) protocol. **D** Total body mass. **E** Total fat mass. **F** Total lean mass (WT *n* = 8, KO *n* = 9). **G-J** Indirect calorimetry data represented as the average for light and dark cycles during last 48 h of data recording. **G** Energy expenditure. **H** Food intake. **I** Locomotor activity. **J** Respiratory exchange ratio (RER) (WT *n* = 6, KO *n* = 6). **K** Glucose tolerance test (GTT). **L** Area under the curve quantification for the GTT. **M** Fasting glucose levels. **N** Insulin tolerance test (ITT). **O** Area under the curve quantification for the ITT (WT *n* = 8, KO *n* = 9). Data are expressed as means ± SEM. Significant differences were determined by Student’s *t*‐test, using a significance level of *p* ≤ 0.05. ***p ≤ 0.0001

To determine whether sex steroid hormones contribute to the leaner phenotype observed in female mice, we performed bilateral ovariectomy surgeries in 5-week-old mice (Supplementary Fig. 7 A) followed by 12 weeks of HFD feeding. Successful ovariectomy surgery was confirmed by reduced uterus weight in ovariectomized (OVX) females relative to sham-operated mice (Supplementary Fig. 7B). *Gdf15* mRNA levels were reduced in BAT of KO mice (Fig. 7A), whereas GDF15 serum levels were unchanged (Fig. 7B). Both WT and KO OVX females experienced similar weight gain when fed HFD (Fig. 7C and D). Consistently, body composition analysis showed unchanged total fat mass (Fig. 7E) and lean mass (Fig. 7F) between OVX-WT and OVX-KO mice. Indirect calorimetry showed no differences in energy expenditure (Fig. 7G and supplementary Fig. 7 C), food intake (Fig. 7H), locomotor activity (Fig. 7I) or respiratory exchange ratio (Fig. 7J) between genotypes. Glucose homeostasis as assessed by GTT (Fig. 7K and L) and fasting glucose levels (Fig. 7M) were similar between WT and KO females. Likewise, ITT was comparable between genotypes (Fig. 7N and O). Interestingly, beyond its metabolic effects, our data also reveal that male mice exhibit higher circulating GDF15 levels than females (Fig. 1A and B) consistent with clinical findings (Lau et al. 2019). Interestingly, ovariectomy abolishes this male–female difference, raising GDF15 levels in BAT and in the serum of female WT mice to those observed in males (Supplementary Fig. 7D and E). *Esr1*, the gene encoding the estrogen receptor α (ERα), has been implicated in changes in metabolic homeostasis. Indeed, reduced ERα action in brown adipocytes impairs mitochondrial function, promotes increased adiposity, and disrupts metabolic homeostasis (Zhou et al. 2020). Therefore, we checked the expression level of *Esr1* in male and female mice after 12 weeks of HFD feeding. Here, we show that *Esr1* expression in female BAT remained unchanged after 12 weeks of HFD feeding (Supplementary Fig. 7 F) and were unnafected by OVX (Supplementary Fig. 7G). In contrast, male GDF15 KO mice has lower *Esr1* expression in BAT compared to their littermate control after 12 weeks of HFD feeding (Supplementary Fig. 7H). Together, our data suggest a role for sex-steroid hormones in driving the metabolic changes contributing to leanness in KO female mice and in regulating GDF15 levels.

### Cell autonomous effect of GDF15 in brown adipocytes

GDF15 has been shown to be released by brown adipocytes in vitro in response to thermogenic activation. GDF15 released by brown adipocytes was suggested to target macrophages likely mediating downregulation of local inflammatory response in BAT (Campderros et al. 2019). Here, we tested if GDF15 has any cell autonomous effects in thermogenic gene expression in mouse primary brown adipocytes. Primary brown adipocytes were isolated from WT and GDF15 BKO mice (Supplementary Fig. 7I). Interestingly, reduced *Gdf15* levels were associated with a significant increase in thermogenic genes. Together these findings indicate that endogenous GDF15 have cell-autonomous effects in mouse brown adipocytes, warranting further investigation of GDF15 signaling in these cells.

## Discussion

GDF15 has recently emerged as an endocrine regulator of energy metabolism and glucose homeostasis with therapeutic potential for the treatment of obesity and associated metabolic diseases (Mullican et al. 2017; Tsai et al. 2018; Breit et al. 2017). Although studies suggest that the liver is the main source of circulating GDF15 in mice fed obesogenic diets (Xie et al. 2022; Patel et al. 2022), the role of BAT-derived GDF15 on systemic metabolic homeostasis is less clear. Our previous study demonstrated that mitochondrial stress in BAT leads to secretion of GDF15 as a batokine, contributing to resistance to DIO and improvements in glucose homeostasis, suggesting BAT-derived GDF15 may contribute to changes in systemic metabolism (Pereira et al. 2021; Jena et al. 2023). Therefore, we tested the hypothesis that BAT-derived GDF15 contributes to regulate systemic metabolic homeostasis in response to HFD feeding in mice.

Surprisingly, our data show that *Gdf15* expression in BAT regulates diet-induced weight accrual and adiposity in a sex-dependent manner. While GDF15 BKO male mice were susceptible, female mice were resistant to DIO. Multiple studies investigating the role of GDF15 in the regulation of appetite and metabolism have been performed exclusively in male rodents (Patel et al. 2019; Patel et al. 2022; Tsai et al. 2018; Miyake et al. 2021; Lu et al. 2023; Tsai et al. 2019), with only a few also addressing females (Tran et al. 2018; Macia et al. 2012; Sjoberg et al. 2023). Relative to WT mice, both male and female *Gdf15* global kockout mice have been shown to have exacerbated weight gain when fed HFD, with males having a more robust phenotype relative to females (Tran et al. 2018). However, there is evidence showing sex-specific GDF15 actions with regard to lipid metabolism in mice. One study reported that female mice ubiquitously expressing human GDF15 lost a higher percentage of total WAT mass compared to their male littermates, which was associated with changes in lipolytic gene expression but not in food intake (Chrysovergis et al. 2014). Moreover, a recent study revealed that *Gdf15* ^−/−^ female mice have greater WAT remodeling compared to male mice even under baseline conditions (Igual-Gil et al. 2024). Interestingly, recombinant GDF15 treatment in mice fed HFD reduced food intake and attenuated weight gain and adiposity in males, whereas female mice were resistant to GDF15’s action, suggesting that GDF15 has sex-specific effects on energy homeostasis (Jeromson et al. 2024). Noteworthy, a recent study investigating GDF15’s role in atherosclerosis, showed significant sex dimorphism with male mice displaying greater weight gain, whereas females were refractory to these effects. Following ovariectomy, female metabolic phenotype mirrored those of males, suggesting female sex hormones play a pivotal role in modulating these metabolic changes (Artz et al. 2016; Davezac et al. 2025), as also shown here.

Our data suggest that *Gdf15* deletion selectively in BAT likely induces compensatory mechanisms, including *Gdf15* regulation and metabolic adaptations in other tissues, as well as hormonal regulation, contributing to our sex-dimporphic phenotype. Indeed, we see increased *Gdf15* levels in the liver of GDF15 BKO male mice, while no changes were detected in females. Moreover, we show that ovariectomy abrogates the metabolic protection observed in females. It is well reported that, despite the growing prevalence of obesity in both sexes, clinical manifestations and complications differ between sexes, with premenopausal women exhibiting relative protection (Virani et al. 2020; Colafella and Denton 2018; Koceva et al. 2024). Importantly, BAT function and the capacity for WAT browning are sexually dimorphic, favoring females (Kim et al. 2016; Lee et al. 2016). Moreover, evidence from rodent models to humans suggest that sex differences in BAT function contribute to the metabolic protection observed in females, via poorly understood mechanisms (Lundgren et al. 2008; Macotela et al. 2009; Tran et al. 2010; Takeuchi et al. 2025). Female mice on HFD have greater metabolic flexibility to adapt to higher energy consumption, including mitochondrial adaptations in BAT, resulting in improved energy homeostasis (MacCannell et al. 2021). Our results suggest an underappreciated but recently suggested link between GDF15 and sex-steroid hormones might contribute to such adaptations in females. Several studies have shown that sex hormones regulate *Gdf15* expression, influencing its role in metabolism, atherosclerosis, and mood. For instance, testosterone and estradiol, particularly in combination, can suppress GDF15 secretion and expression, potentially through receptor-mediated pathways in the liver and other tissues. Moreover, GDF15 deficiency is associated with sex-dimorphism in metabolic and cardiovascular diseases (Guillaume et al. 2019; Faubion et al. 2020; Liu et al. 2019; Pena-Leon et al. 2024). Our study contributes to this growing literature, highlighting the need for sex-specific considerations in therapies targeting GDF15, and placing *Gdf15* regulation in BAT at the interplay between metabolic flexibility and sex-steroid hormone actions during DIO.

Another important observation in our study is that deletion of *Gdf15* in BAT led to significant systemic effects despite no major changes in GDF15 serum levels under ad libitum-fed conditions. Our findings suggest that in addition to its central effects, GDF15 might also mediate autocrine/paracrine signaling in BAT and have cell-autonomous effects in brown adipocytes, contributing to systemic metabolic homeostasis in mice during DIO. Although GFRAL has been the only receptor for GDF15 thoroughly validated to date, it has been proposed that GDF15 may mediate its effects through additional targets, especially in peripheral tissues, in a GFRAL-independent manner (Artz et al. 2016; Zhang et al. 2023; Gao et al. 2021; Li et al. 2018; Patsalos et al. 2022; Wang et al. 2021; Kempf et al. 2011). However, GFRAL-independent effects are still controversial as several concerns have been raised, including GDF15 dosing, potential contamination with TGF-β in in vitro studies (Olsen et al. 2017) and the possibility that GFRAL may be expressed in locations other than the hindbrain, as recently suggested (Mullican et al. 2017; Fichtner et al. 2024; Xie et al. 2023). Because our studies were conducted primarily in vivo and investigated GDF15 in the context of its endogenous regulation, the first two concerns do not apply to our circumstances. However, we cannot discard the possibility that GFRAL, like GDF15, may be induced in certain tissues, including BAT, in response to stress and/or pathological conditions. Previous studies have also indicated that the cleavage process of pro-GDF15 occurs in the nucleus, and it can influence signaling pathways including the SMAD and the Hippo signaling pathways, thereby regulating transcription within the nucleus (Li et al. 2024). Athough beyond the scope of the present study, identification and validation of potential novel GDF15 receptors and/or signaling mechanisms within BAT and other tissues will be critical to advance our understanding of GDF15 biology and pharmachology.

*Gdf15* deletion in BAT of male mice resulted in exacerbated weight gain and increased fat deposition, likely contributing to fibro-inflammation (Pellegrinelli et al 2023). Noteworthy, we did not detect any changes in food intake, energy expenditure or locomotor activity that could explain the changes in weight gain in male mice. This is a limitation of our study likely due to relatively small number of mice reducing statistical power. Another possibility is that, because indirect calorimetry measurements were performed under thermoneutral conditions, small changes related to thermoregulation that might have been present at room temperature were no longer detactable at thermoneutrality. Regardless of the mechanisms driving weight gain, the induction of inflammatory markers in BAT of KO mice in the absence of changes in GDF15 seum levels suggest GDF15 might play a local anti-inflammatory role during obesity. Interestingly, in addition to its effects on food intake, a recent study suggested that GDF15 released by brown adipocytes in response to increased thermogenic activity targets macrophages and may mediate downregulation of local inflammatory pathways (Campderros et al. 2019). Moreover, *Gdf15* ^−/−^ mice have increased markers of inflammation and fibrosis in the liver even when fed regular chow, supporting the idea that GDF15 may exert local effects to mitigate inflammation and extracellular matrix (ECM) remodeling (Jurado-Aguilar et al. 2024). Although we have not measured inflammatory markers at the protein level or directly assessed macrophage infiltration in BAT, based on the lack of changes in GDF15 serum levels following HFD feeding, we speculate similar mechanisms might be at play in our model that might contribute to the overall metabolic phenotype. Indeed, BAT inflammatory state has been shown to have a significant effect on glucose homeostasis, with research showing that excessive inflammation can disrupt metabolic function and contribute to insulin resistance (Villarroya et al. 2018). Another study recently showed that obesity-related fibro-inflammation compromises BAT ECM remodeling and functionality, contributing to obesity-associated metabolic disturbances (Pellegrinelli et al. 2023). Future time-course studies will be required to dissect whether GDF15 deletion plays any causal role on diet-induced increases in inflammatory and ECM markers in BAT even prior to changes in body weight or if exacerbated inflammation is just a function of increased weight gain and adiposity in male KO mice.

## Conclusions

In conclusion, we show that *Gdf15* expression in BAT plays an important role in regulating weight gain and adiposity during DIO in a sex-dependent manner and undescore the importance of rigorously addressing sex differences in GDF15 biology. Our data also has important clinical implications as GDF15-targeted therapies are currently being investigated for the treatment of obesity and cachexia. Importantly, in cachexia, high systemic levels of tumor- or injury-derived GDF15 promote anorexia, muscle wasting, and white adipose tissue loss via central GFRAL/RET receptor signaling (Groarke et al. 2024; Ling et al. 2023; Kim-Muller et al. 2023). Our work underscores the importance of considering endogenous beneficial functions of GDF15 in brown adipose tissue when developing GDF15-targeted therapies for cachexia, highlighting the need for strategies that selectively inhibit pathogenic pathways without disrupting physiological pathways that regulate systemic metabolic homeostasis. Our study also suggests that sex and hormonal status must be carefully considered in future therapeutic strategies and reinforce the idea that metabolic consequences of GDF15 modulation may differ substantially between sexes. Noteworthy, in addition to its central effects on food intake, our results suggest that GDF15 might also exert local effects through autocrine/paracrine signaling contributing to BAT function with significant implications for systemic metabolic health.

## Supplementary Information

Supplementary Material 1.

Supplementary Material 2.

## Data Availability

All datasets generated and/or analyzed during the current study are presented in the article, or are available from the corresponding author upon reasonable request.

## Abbreviations

GFRAL: Glial-derived neurotrophic factor receptor α family-like specific receptor
HFD: High-fat diet
BAT: Brown adipose tissue
iWAT: Inguinal white adipose tissue
DIO: Diet-induced obesity
BKO: Brown adipose tissue knockout
GTT: Glucose tolerance tests
ITT: Insulin tolerance tests
GC–MS: Gas chromatography-mass spectrometry
NMR: Nuclear magnetic resonance
SVF: Stromal vascular fraction
TG: Triglyceride
AUC: Area under the curve
PBS: Phosphate-buffered saline
FBS: Fetal bovine serum
EtOH: Ethanol
DMEM: Dulbecco’s modified eagle’s medium
OxPhos: Oxidative phosphorylation
*Tbp*: TATA-binding protein
*Gdf15*: Growth differentiation factor 15
*RT-PCR*: Real time polymerase chain reaction
ANOVA: Analysis of variance
BSA: Bovine serum albumin
T3: Triiodothyronine
OVX: Ovariectomy
HEPES: 4-(2-Hydroxyethyl)-1-piperazineethanesulfonic acid
NaCl: Sodium chloride
MgCl_2_: Magnesium chloride
EGTA: Ethylene glycol-bis(β-aminoethyl ether)-*N*,*N*,*N’*,*N*′-tetraacetic acid
KCl: Potassium chloride
K-MES: MES potassium salt
KH_2_PO4: Monobasic potassium phosphate
*Ucp1*: Uncoupling protein 1
*Prdm16*: PR domain containing 16
*Dio2*: Iodothyronine deiodinase 2
*Cpt1β*: Carnitine palmitoyl transferase 1B
*Elovl6*: ELOVL fatty acid elongase 6
*Ppargc1α*: Peroxisome proliferator-activated receptor gamma coactivator 1-alpha
*Dgat*: Diacylglycerol O-acyltransferase
*Gpat*: Glycerol-3-phosphate acyltransferase
*Ppar-γ*: Peroxisome proliferator-activated receptor gamma
*Timp2*: Tissue inhibitor of metalloproteinases 2
*Col1a1*: Collagen type I alpha 1 chain
*Col1a2*: Collagen type I alpha 2 chain
*F4/80*: EGF-like module-containing mucin-like hormone receptor-like 1
*Mcp1*: Monocyte Chemoattractant Protein-1
*Pnpla3*: Patatin-like phospholipase domain-containing protein 3
*Fasn*: Fatty acid synthase
*Ppar-α*: Peroxisome proliferator-activated receptor alpha
*Lcad*: Acyl-CoA dehydrogenase, long chain
*Acadm*: Acyl-Coenzyme A dehydrogenase medium chain
*Acaca*: Acetyl-CoA carboxylase
*Esr1*: Estrogen receptor 1
