# Supplementary material for: Gdf15 expression in thermogenic adipocytes regulates diet-induced weight gain in a sex-dependent manner
Source: Mol Med. 2026 May 18;32:108. doi: 10.1186/s10020-026-01505-5 (PMC13352486; doi:10.1186/s10020-026-01505-5)
Supplement: Supplementary file 1 — Supplementary Material 1. [file 10020_2026_1505_MOESM1_ESM.docx]

**Supplementary Materials and Methods**

***Gdf15* expression in thermogenic adipocytes regulates energy homeostasis in a sex-dependent manner**

Jena J, et al.

**Cell culture and treatments:**

**Brown adipocyte and stromal vascular fraction (SVF) isolation:**

Primary brown adipocytes were isolated from iBAT depots of GDF15 BKO mice as previously described (1). Briefly, adipose tissues were minced and digested in 1 mg/mL liberase (Roche, Switzerland) in Krebs-Ringer bicarbonate buffer, KRB (Sigma, St. Louis, MO) in a 37 °C water bath with agitation for 30 minutes, followed by filtration through nylon mesh. The samples were centrifuged at 500×g for 10 minutes at 10°C. The floating mature adipocytes (BA) and SVF were collected for further analysis.

**Primary adipocyte isolation and culture:**

Primary adipocytes were isolated from BAT of 3-week-old GDF15 BKO or WT control mice. Cells were grown and differentiated as previously described (2, 3). Briefly, BAT was dissected and digested in 0.4% Type I collagenase (Worthington, NJ) in Krebs-Ringer Bicarbonate Buffer (Sigma, St. Louis, MO). Digested tissue was then placed into a prewarmed shaker (37 °C) and allowed to shake at 150 rpm for 1h, followed by centrifugation for 5 min at room temperature. The supernatant was discarded and the pellet was washed and centrifuged three times with preadipocyte growth media (DMEM F/10) containing 10% fetal bovine serum (FBS), 50U/ml Penicilin and 50 ug/ml streptomycin. After the final wash, the preadipocyte growth media was removed and the pellet was resuspended in fresh preadipocyte growth media. The resuspended cells were filtered through a 40μm nylon cell strainer and cells were plated in 6-well plates. Finally, isolated pre-adipocytes were incubated and allowed to grow until 80% confluence, when they were then differentiated into mature adipocytes for 7 days in DMEM F/10 containing 0.4 μg/ml insulin (Thermo Fisher, #12585014), 2nm T3 (Sigma-Aldrich, MO, USA #T2877), 0.5 μM dexamethasone (Sigma-Aldrich, MO, USA, #D1756), 0.5 μM rosiglitazone (Cayman chemicals, Ann Arbor, USA, #71740), 60 uM Indomethacin (Sigma-Aldrich, MO, USA, # I7378 ) and 250 uM 3-isobutyl-1-methyl-xanthine (Sigma-Aldrich, MO, USA, #I5879) for 4 days. Following differentiation into mature adipocytes, cells were put in maintenance media (DMEM F/10 containing 0.4 μg/ml insulin, 2nM T3 and 0.1 uM Rosiglitazone) until used for experiment (day 7). Cells were washed with ice-cold PBS and harvested for subsequent analysis.

**12-hour fasting glucose measurements:**

Twelve-week-old male GDF15 BKO mice and their WT littermate controls were fasted for 12 hours (7 pm to 7 am). Following fasting, blood glucose levels were measured using glucometer.

**Ovariectomy surgeries**

Mice were placed dorsal side up on the operating area. Two bi-lateral incisions were made (~1 cm) lateral to the midline and junction of the spine and pelvis. Dumont forceps were used to blunt dissect through the muscle wall to expose the ovarian fat pad. Ovaries and a small part of the uterus were externalized, clamped beneath the oviduct,absorbable suture was tied below the clamp, and ovaries excised above the clamp. Hemostasis was ensured and the uterus returned to its position within the pelvic cavity. Incisions were closed with suture and analgesia administered.

**RNA extraction and quantitative RT-PCR**

Total RNA was extracted from tissues using TRIzol reagent (Invitrogen, Waltham, MA) and purified with the RNeasy kit (QIAGEN Inc, Germantown, MD). RNA concentration was determined by measuring absorbance at 260 and 280 nm with a spectrophotometer (NanoDrop 1000, NanoDrop products, Wilmington, DE). Total RNA (0.5 µg) was reverse transcribed using High-Capacity cDNA Reverse Transcription Kit (Applied Biosystems, Waltham, MA), followed by quantitative RT-PCR using SYBR Green (Applied Biosystem, Life Technologies, Warrington, UK) as previously described (2). Samples were loaded in a 384-well plate in triplicate, and real-time polymerase chain reaction was performed with an Applied Biosystems QuantStudio 7 Flex (*QS-7 Flex*). The following cycle profile was used: 1 cycle at 95˚C for 10 min; 40 cycles of 95˚C for 15 s; 59˚C for 15 s, 72˚C for 30 s, and 78˚C for 10 s; 1 cycle of 95˚C for 15 s; 1 cycle of 60˚C for 15 s; and 1 cycle of 95˚C for 15 s. Collected data was analyzed using Design and Analysis Software v2.3. Data were normalized to *Tbp* expression and results are shown as relative mRNA expression in fold change. qPCR primers were designed using Primer‐Blast or previously published sequences. Utilized primers are listed in Supplemental Table 1.

**RNA Sequencing in BAT**For bulk RNA sequencing, libraries were prepared using Illumina’s (San Diego, CA) TruSeq Stranded mRNA Library Prep and sequenced on a NovaSeq 6000. Single-end reads were quantified using Kallisto (v0.46.2) and differential expression analysis performed with DESeq2 (v1.42.0). Pathway analysis was performed using Qiagen’s Ingenuity Pathway Analysis. Plots were generated using the packages ggplot2 and pheatmap in the R version (v4.3.0) or GraphPad Prism Software (La Jolla, CA).

**Metabolomics**

***Sample preparation:*** Whole BAT tissue was disrupted using bead mill homogenization in 18:1 (µl:mg wet tissue weight) ice-cold 2:2:1 methanol/acetonitrile/water extraction buffer containing a mixture of internal standards (D4-citric acid, D4-succinic acid, D8-valine, and U13C-labeled glutamine, glutamic acid, lysine, methionine, serine, and tryptophan; Cambridge Isotope Laboratories, Tewksbury, MA). Homogenates were rotated for 1 h at -20˚C, then centrifuged for 10 minutes at 21,000 x g, and 150 µl of cleared metabolite extracts were transferred to autosampler vials and dried using a SpeedVac vacuum concentrator (Thermo Fisher Scientific, Waltham, MA). Dried metabolite extracts were reconstituted in 30 μl of 11.4 mg/ml methoxyamine (MOX) in anhydrous pyridine, vortexed for 5 minutes, and heated for 1 h at 60˚C. Next, 20 μl of N,O-Bis(trimethylsilyl)trifluoroacetamide (TMS) was added to each sample, samples were vortexed for 1 min, and heated for 30 min at 60˚C.

***GC-MS method:*** Derivatized samples were analyzed by GC-MS. 1 μl of derivatized sample was injected into a Trace 1300 GC (Thermo Fisher) fitted with a TraceGold TG-5SilMS column (Thermo Fisher) operating under the following conditions: split ratio = 20:1, split flow = 24 μl/min, purge flow = 5 ml/minute, carrier mode = constant flow, and carrier flow rate = 1.2 ml/min. The GC oven temperature gradient was as follows: 80˚C for 3 min, increasing at a rate of 20˚C/min to 280˚C, and holding at 280˚C for 8 min. Ion detection was performed by an ISQ 7000 mass spectrometer (Thermo Fisher) operated from 3.90 to 21.0 min in EI mode (-70eV) using select ion monitoring (SIM). Raw data were analyzed using TraceFinder 5.1 (Thermo Fisher). Metabolite identification and annotation required at least two ions (target + confirming) and a unique retention time that corresponded to the ions and retention time of a reference standard previously determined in-house. A pooled-sample generated prior to derivatization was analyzed at the beginning, at a set interval during, and at the end of the analysis run to correct peak intensities using the NOREVA tool (4). NOREVA corrected data were then normalized to the sum total of signal per sample to control for extraction, derivatization, and/or loading effects.

**Mitochondrial isolation**

Mitochondrial fraction was isolated from BAT, as previously described (2). Briefly, tissue was excised, rinsed in ice-cold PBS, and maintained in ice-cold isolation buffer (500 mM EDTA, 215 mM D-mannitol, 75 mM sucrose, 0.1% free fatty acid bovine serum albumin (BSA), 20 mM HEPES, pH 7.4 with KOH) until ready for homogenization. Bradford assay was performed to determine protein concentration.

**Oxygen consumption**

Mitochondrial oxygen consumption rates were assessed in 50 µg of mitochondrial protein using the Oroboros O_2_K Oxygraph system (Oroboros Instruments, Innsbruck, Austria) in 2.5 mL of buffer Z containing (in mmol/l): 30 KCl, 105 K-MES, 5 MgCl_2_-6H_2_O, 1 EGTA, 10 KH_2_PO_4_ and 2.5 mg/ml BSA (pH 7.4 adjusted). Substrates were added to the assay buffer at the following final concentrations: pyruvate at 5 mM, malate at 2.5 mM, palmitoyl carnitine at 4 mM and succinate stimulation at 10 mM.

**Western blot analysis**

Approximately 30-50 mg frozen tissue was homogenized in 200 μl of lysis buffer comprising (mmol/l) 50 HEPES, 150 NaCl, 10% glycerol, 1% Triton X-100, 1.5 MgCl_2_, 1 EGTA, 10 sodium pyrophosphate, 100 sodium fluoride, and 100 μmol/l sodium vanadate. Just before use, HALT protease/phosphatase inhibitors (Thermo Fisher) were added to the lysis buffer, and samples were processed using the Tissue Lyser II (Qiagen). Tissue lysates were resolved on SDS–PAGE and transferred to nitrocellulose membranes (Millipore Corp., Billerica, MA). Membranes were incubated overnight with primary antibodies and for 1 h with secondary antibodies at room temperature. Data was analyzed using Image Studio Lite (LI-COR Technologies, Lincoln. NE) and was normalized by the specified loading controls. Data represented as arbitrary units of optical density (OD).

**Antibodies**

Primary antibodies: GAPDH (1:1,000, Cell Signaling Technology, Danvers, MA, #2118), UCP1 (1:1,000, Abcam, Boston, MA, #Ab10983), PGC-1α Antibody (4A8) (1:500, Santa Cruz Biotechnology, Dallas, TX, # sc-517380), phosphorylated eIF2α serine 51 (1:1,000, Cell Signaling Technology, #3597), total eIF2α (1:500, Santa Cruz Biotechnology, Dallas, TX, #SC81261), β-actin (1:1,000, Sigma Aldrich, St. Louis, MO, # A2066), HSL (1:1,000, Cell Signaling Technology, #4107), phospho-HSL (1:1,000, Cell Signaling Technology, #4139), ATGL (1:1,000,Cell Signaling Technology, #2138), FASN (1:1,000, Cell Signaling Technology, #3180), Total OXPHOS Rodent WB Antibody Cocktail (1:1,000, Abcam, #Ab110413). GDF15 antibody (1:1000, Abcam, #Ab106006). Secondary antibodies: IRDye 800CW anti‐mouse (1:10,000, LI‐COR, Lincoln, NE, #925‐32212) and Alexa Fluor anti‐rabbit 680 (1:10,000, ThermoFisher, #A27042). Fluorescence was quantified using the LiCor Odyssey imager.

**Supplementary Figure Legends**

**Supplementary Figure 1: Baseline assessment of GDF15 BKO male mice.** Data collected on 6-week-old male GDF15 BKO (KO) mice and their WT littermate controls fed regular chow diet (WT n=6, KO n=6). **A** Representative immunoblots and **B** Respective densitometric quantification of GDF15 protein in BAT of male mice normalized to Ponceau red staining (WT n=4, KO n=6). OD: optical density. **C, D** *Gdf15* mRNA expression in isolated brown adipocytes and SVF of 12-week-old GDF15 BKO and WT mice (WT n=3, KO n=5). **E-K** Metabolic data in 6-week-old males (WT n=9, KO n=10). **E** Body mass. **F** Total lean mass. **G** Total fat mass. **H** Glucose tolerance test (GTT). **I** Area under the curve quantification for GTT (WT n=10, KO n=11). **J** Fasting glucose levels in 6-week-old males (WT n=10, KO n=11). **K** Fasting glucose levels (12 hours) 12-week-old male mice (WT n=8, KO n=6). Data are expressed as means ± SEM. Significant differences were determined by Student’s *t*‐test or Mann-Whitney test, using a significance level of *p* ≤0.05. **p*≤ 0.05; ***p*≤ 0.01.

**Supplementary Figure 2: Assessment of GDF15 BKO male mice after 12 weeks on HFD.** Data collected in male GDF15 BKO (KO) and WT littermate control fed HFD for 12 weeks. **A** Relative *Gdf15* mRNA levels in BAT normalized to *Tbp* (WT n=5, KO n=7). **B** Fast GDF15 serum levels (WT n=6, KO n=5). **C** Relative *Gdf15* mRNA levels in iWAT normalized to *Tbp* (WT n=5, KO n=7). **D** Relative *Gdf15* mRNA levels in liver normalized to *Tbp* (WT n=5, KO n=6). **E** Regression plot for energy expenditure as a function of body weight (WT n=5, KO n=6). **F** Relative mRNA expression of fatty acid oxidation (FAO) genes in BAT normalized to *Tbp* (WT n=5, KO n=7)*.* **G** Relative mRNA expression of lipid metabolism genes in BAT normalized to *Tbp* (WT n=5, KO n=7). **H** Representative immunoblots of FASN, phosphorylated HSL (pHSL) normalized to total HSL and ATGL in BAT normalized to Ponceau (WT n=5, KO n=7). **I** Densitometric quantification of pHSL/HSL, ATGL and FASN. **J** Immunoblot and **K** respective densitometric quantification of OXPHOS protein in BAT (WT n=5, KO n=7). **L** UCP1 and PGC1α protein levels normalized to β-Actin in iWAT and **M** respective densitometric quantification (WT n=5, KO n=7). **N** Relative mRNA expression of thermogenic genes normalized to *Tbp* in iWAT. **O** Relative mRNA expression of fatty acid oxidation genes normalized to *Tbp* in iWAT (WT n=5, KO n=7). Data are expressed as means ± SEM. Significant differences were determined by Student’s *t*‐test or ANCOVA analysis, using a significance level of *p* ≤0.05. **p*≤ 0.05; ****p*≤ 0.001.

**Supplementary Figure 3: Global transcriptomic and metabolomic analysis in BAT of GDF15 BKO male mice fed HFD.** Data collected in 18-week-old male GDF15 BKO (KO) and WT littermate controls fed high-fat diet for 12 weeks. **A** Heatmap of the top 50 differentially expressed genes in GDF15 BKO mice (WT n=4, KO n=4). **B** Relative mRNA expression of fibrosis markers in BAT normalized by *Tbp* (WT n=5, KO n=7). **C** Relative mRNA expression of macrophage markers in BAT normalized by *Tbp* (WT n=5, KO n=7). **D** Enrichment analysis of the top 25 metabolites. Highlighted in red are lipid metabolism processes, including fatty acids biosynthesis, elongation, and beta oxidation. Data are expressed as means ± SEM. Significant differences were determined by Student’s *t*‐test, using a significance level of *p*≤0.05. **P*≤0.05; ***p*≤0.01.

**Supplementary Figure 4:** **Baseline assessment of GDF15 BKO female mice.** Data collected on 6-week-old female GDF15 BKO (KO) mice and their WT littermate controls fed regular chow diet (WT n=10, KO n=12). **A** Relative *Gdf15* mRNA levels in brain normalized to *Tbp*. **B** Relative *Gdf15* mRNA levels in kidney normalized to *Tbp*. **C** Relative *Gdf15* mRNA levels in liver normalized to *Tbp*. **D-I** Metabolic data in 6-week-old females (WT n=10, KO n=12). **D** Body mass. **E** Total lean mass. **F** Total fat mass. **G** Glucose tolerance test (GTT). **H** Area under the curve quantification for the GTT (WT n=15, KO n=10). **I** Fasting glucose levels in 6-week-old females (WT n=12, KO n=10). Data are expressed as means ± SEM. Significant differences were determined by Student’s *t*‐test, using a significance level of *p* ≤ 0.05.

**Supplementary Figure 5: Assessment of GDF15 BKO female mice after 12 weeks on HFD.** Data collected in Female GDF15 BKO (KO) and WT littermate control fed HFD for 12 weeks. **A** Relative *Gdf15* mRNA levels in BAT normalized to *Tbp* (WT n=6, KO n=6). **B** Fast GDF15 serum levels (WT n=6, KO n=5). **C** Relative *Gdf15* mRNA levels in iWAT normalized to *Tbp* (WT n=6, KO n=6). **D** Relative *Gdf15* mRNA levels in liver normalized to *Tbp* (WT n=5, KO n=6). **E** Regression plot for energy expenditure (EE) as a function of body weight. **F** Relative mRNA expression of fatty acid oxidation (FAO) genes in BAT normalized to *Tbp* (WT n=6, KO n=6)*.* **G** Relative mRNA expression of lipid metabolism genes in BAT normalized to *Tbp* (WT n=6, KO n=6). **H** Representative immunoblots of FASN, phosphorylated HSL (pHSL) normalized to total HSL and ATGL in BAT normalized to Ponceau (WT n=5, KO n=5). **I** Densitometric quantification of pHSL/HSL, ATGL and FASN. **J** Immunoblot and **K** respective densitometric quantification of OXPHOS protein in BAT (WT n=5, KO n=5). **L** UCP1 and PGC1α protein levels normalized to β-Actin in iWAT and **M** respective densitometric quantification (WT n=6, KO n=6). **N** Relative mRNA expression of thermogenic genes normalized to *Tbp* in iWAT. **O** Relative mRNA expression of fatty acid oxidation genes normalized to *Tbp* in iWAT (WT n=6, KO n=6). Data are expressed as means ± SEM. Significant differences were determined by Student’s *t*‐test or ANCOVA analysis, using a significance level of *p* ≤0.05. **p*≤ 0.05; ***p*≤ 0.01; ****p*≤ 0.001.

**Supplementary Figure 6: Global transcriptomic and metabolomic analysis in BAT of GDF15 BKO female mice fed HFD.** Data was collected in 18-week-old female GDF15 BKO (KO) and WT littermate controls fed high-fat diet for 12 weeks. **A** Heatmap showing differentially expressed genes in various pathways (WT n=4, KO n=4). **B** Relative mRNA expression of fibrosis markers in BAT normalized by *Tbp* (WT n=6, KO n=6). **C** Relative mRNA expression of macrophage markers in BAT normalized by *Tbp* (WT n=5, KO n=3 or 6). **D** Protein levels of phosphorylated eIF2α (peIF2α) normalized eIF2α in BAT (WT n=5, KO n=5). **E** Enrichment analysis of the top 25 metabolites. Highlighted in red are TCA cycle and fatty acid oxidation processes. Data are expressed as means ± SEM. Significant differences were determined by Student’s *t*‐test, using a significance level of *p*≤0.05.

**Supplementary Figure 7: Data in ovariectomized mice. A** Representative images of control and ovariectomized uterus (pink squares highlight ovaries). **B** Uterus weight in control and ovariectomized females (WT n=8, KO n=8). **C** Regression plot for energy expenditure (EE) in ovariectomized mice after 12 weeks of HFD feeding. **D** Serum GDF15 levels (male n=6, female n=10, female OVX n=8) **E** Relative *Gdf15* mRNA levels in BAT of WT mice fed HFD normalized to *Tbp* (male n=4, female n=5, female OVX n=6). . **F-H** Relative *Esr1* mRNA levels in BAT of WT versus GDF15 BKO mice fed HFD. **F** GDF15 BKO females, **G** Ovariectomized GDF15 BKO females and **H** GDF15 BKO males. **I** Relative mRNA expression of *Gdf15* and thermogenic genes normalized to *Tbp* in differentiated primary mouse brown adipocytes isolated from GDF15 BKO or WT female mice. Data expressed as means ± SEM. Significant differences determined by One-Way ANOVA, Student’s *t*‐test or Mann-Whitney test, using a significance level of *p* ≤ 0.05. **p*≤ 0.05; ***p*≤ 0.01; ****p*≤ 0.001; *****p*≤ 0.0001.

**Supplementary Table 1:** Primer sequences.

| ***Gene*** | **Forward sequence** | **Reverse sequence** |
| --- | --- | --- |
| *Tbp* | TCTGGAATTGTACCGCAGCTT | CTGCAGCAAATCGCTTGGGA |
| *Gdf15* | GAGAGGACTCGAACTCAGAAC | GACCCCAATCTCACCTCTG |
| *Ucp1* | GTGAAGGTCAGAATGCAAGC | AGGGCCCCCTTCATGAGGTC |
| *Prdm16* | CAGCACGGTGAAGCCATTC | GCGTGCATCCGCTTGTG |
| *Dio2* | AATTATGCCTCGGAGAAGACCG | GGCAGTTGCCTAGTGAAAGGT |
| *Cpt1β* | TGCCTTTACATCGTCTCCAA | AGACCCCGTAGCCATCATC |
| *Elovl6* | TCAGCAAAGCACCCGAAC | AGCGACCATGTCTTTGTAGGAG |
| *Ppargc1α* | GTAAATCTGCGGGATGATGG | AGCAGGGTCAAAATCGTCTG |
| *Dgat* | GGTTCCGTGTTTGCTCTGGCAT | CCACTGACCTTCTTCCCTGTAG |
| *Gpat* | GCAAGCACTGTTACCAGCGATC | TGCAATCAGCCTTCGTCGGAAG |
| *Ppar-γ* | GCCTGTCTGTCGGGATGT | GGCTCCGTGGATTCTCTTG |
| *Timp2* | TGCAGACGTAGTGATCAGAGC | ACTCGATGTCTTTGTCAGGTCC |
| *Col1a1* | AGATGTAGGAGTCGAGGGAC | GGCCTTGGAAACCTTGTGGA |
| *Col1a2* | TTCTGTGGGTCCTGCTGGGAAA | TTGTCACCTCGGATGCCTTGAG |
| *F4/80* | AGGAGCCTGGTACATTGGTG | CTGGGATCCTACAGCTGCTC |
| *Mcp1* | GCTCAGCCAGATGCAGTTAA | TCTTGAGCTTGGTGACAAAAACT |
| *Pnpla3* | AGACAAGGTGCCAGTCAGCCTA | GAGGTTGCAGACTTTGCTCAGG |
| *Fasn* | CTCCGTGGACCTTATCACTA | CTGGGAGAGGTTGTAGTCAG |
| *Ppar-α* | GGGAACTTAGAGGAGAGCCAA | CCATGTTGGATGGATGTGGC |
| *Lcad* | ATGGCAAAATACTGGGCATC | TCTTGCGATCAGCTCTTTCA |
| *Acadm* | CAAGCAGGAGCCCGGATTAG | GAGAGGGAACGGGTACTCCC |
| *Acaca* | CCAGGCCATGTTGAGACGCT | ATCACAGAGCGGACGCCATC |
| *Esr1* | GCCAGAATGGCCGAGAGAG | CCCCATAATGGTAGCCAGAGG |

**References**

1. Sebag SC, Zhang Z, Qian Q, Li M, Zhu Z, Harata M, et al. ADH5-mediated NO bioactivity maintains metabolic homeostasis in brown adipose tissue. Cell Rep. 2021;37(7):110003.

2. Pereira RO, Marti A, Olvera AC, Tadinada SM, Bjorkman SH, Weatherford ET, et al. OPA1 deletion in brown adipose tissue improves thermoregulation and systemic metabolism via FGF21. Elife. 2021;10:e66519.

3. BonDurant LD, Ameka M, Naber MC, Markan KR, Idiga SO, Acevedo MR, et al. FGF21 Regulates Metabolism Through Adipose-Dependent and -Independent Mechanisms. Cell Metab. 2017;25(4):935-44 e4.

4. Li B, Tang J, Yang Q, Li S, Cui X, Li Y, et al. NOREVA: normalization and evaluation of MS-based metabolomics data. Nucleic Acids Res. 2017;45(W1):W162-W70.
