# Supplementary material for: Gdf15 expression in thermogenic adipocytes regulates diet-induced weight gain in a sex-dependent manner
Source: Mol Med. 2026 May 18;32:108. doi: 10.1186/s10020-026-01505-5 (PMC13352486; doi:10.1186/s10020-026-01505-5)
Supplement: Supplementary file 2 — Supplementary Material 2. [file 10020_2026_1505_MOESM2_ESM.pdf]

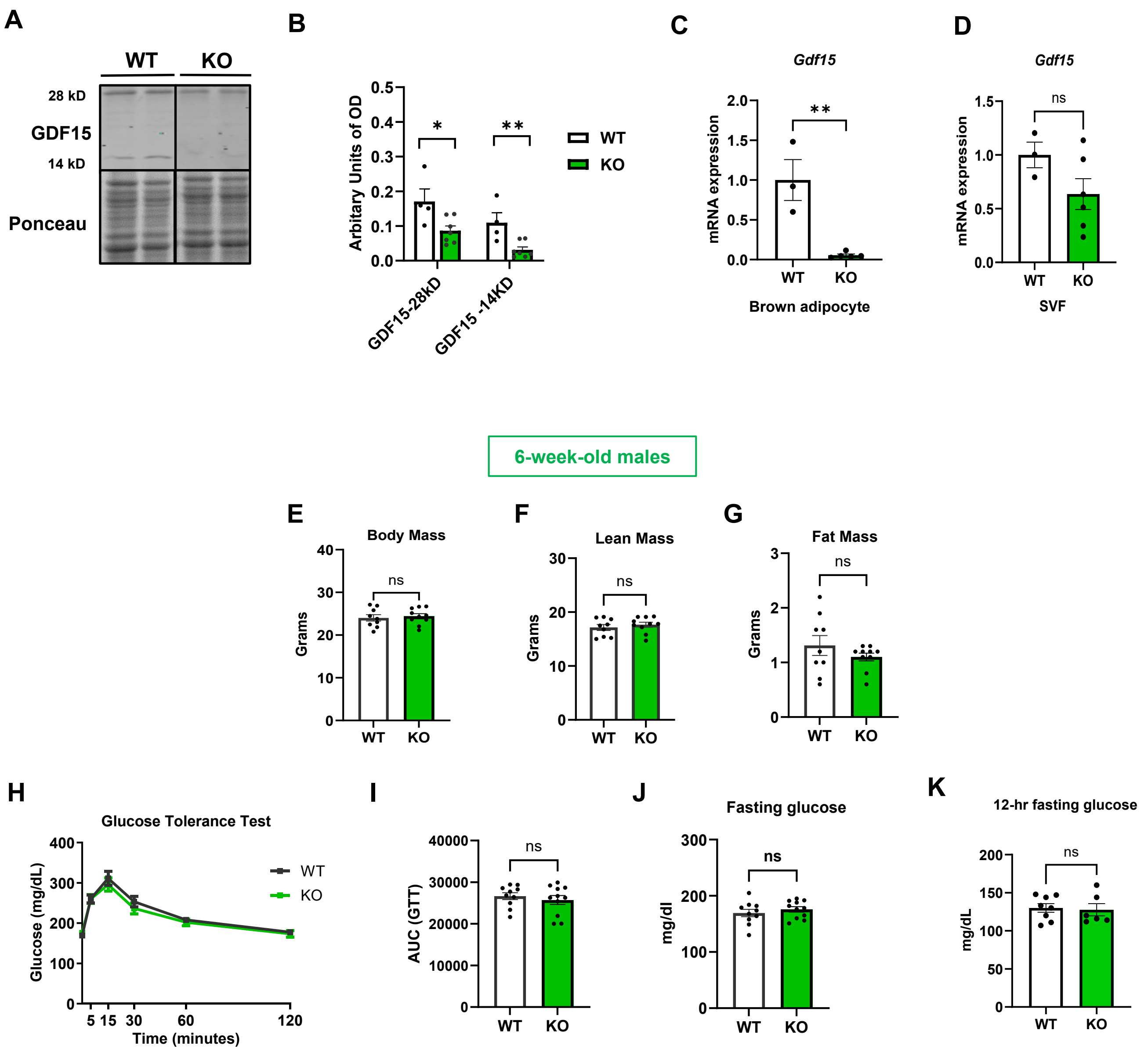

Supplementary Figure 1

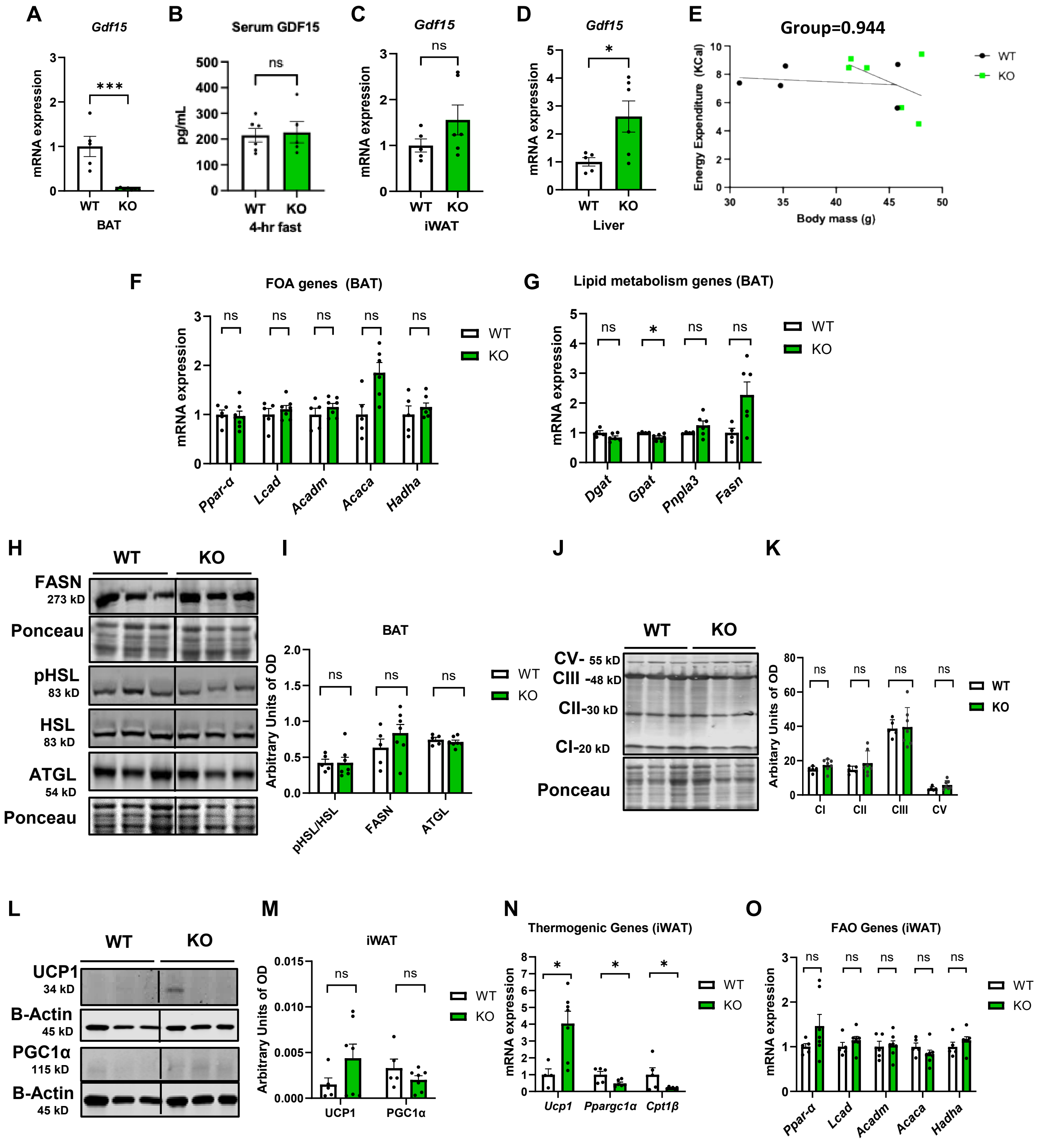

Supplementary Figure 2

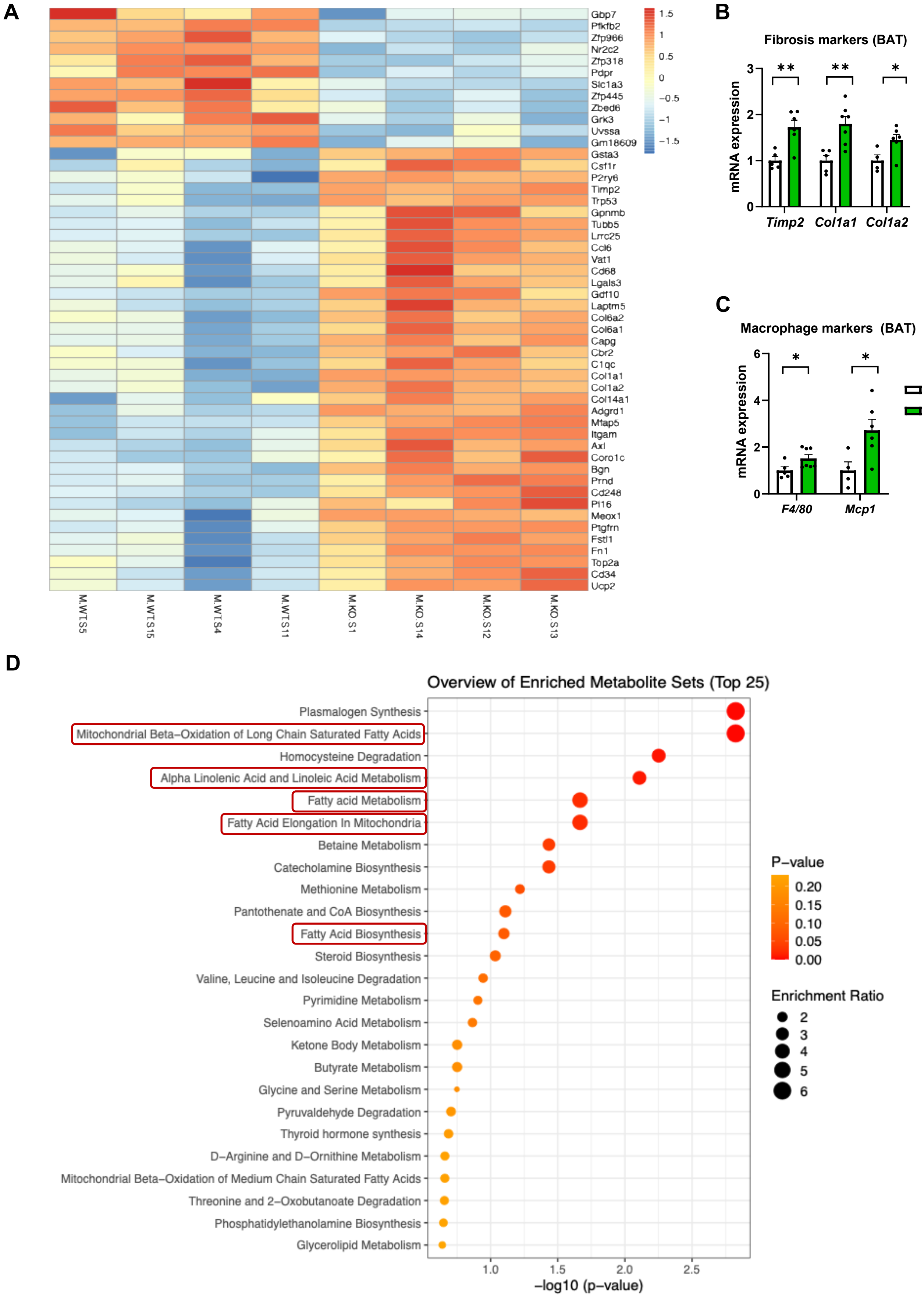

Supplementary Figure 3

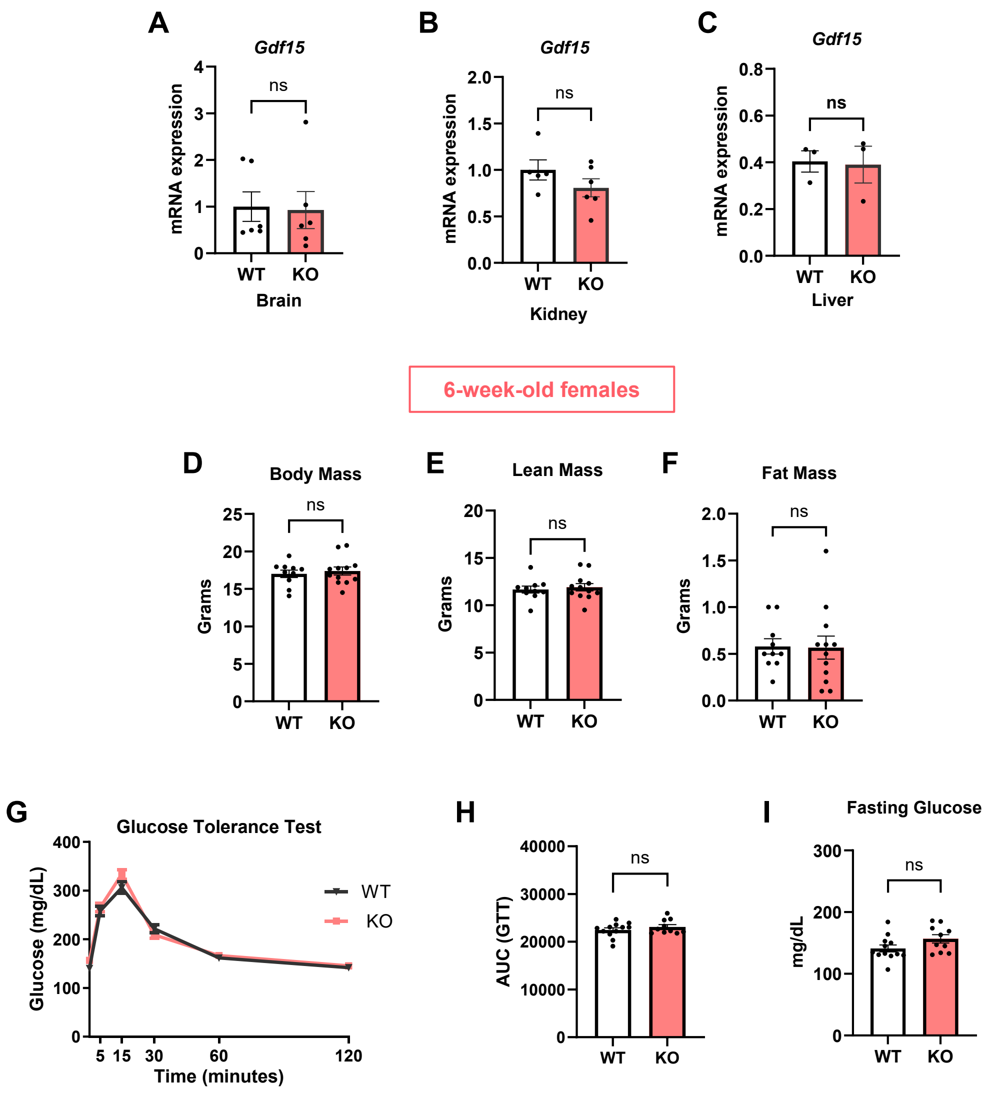

Supplementary Figure 4

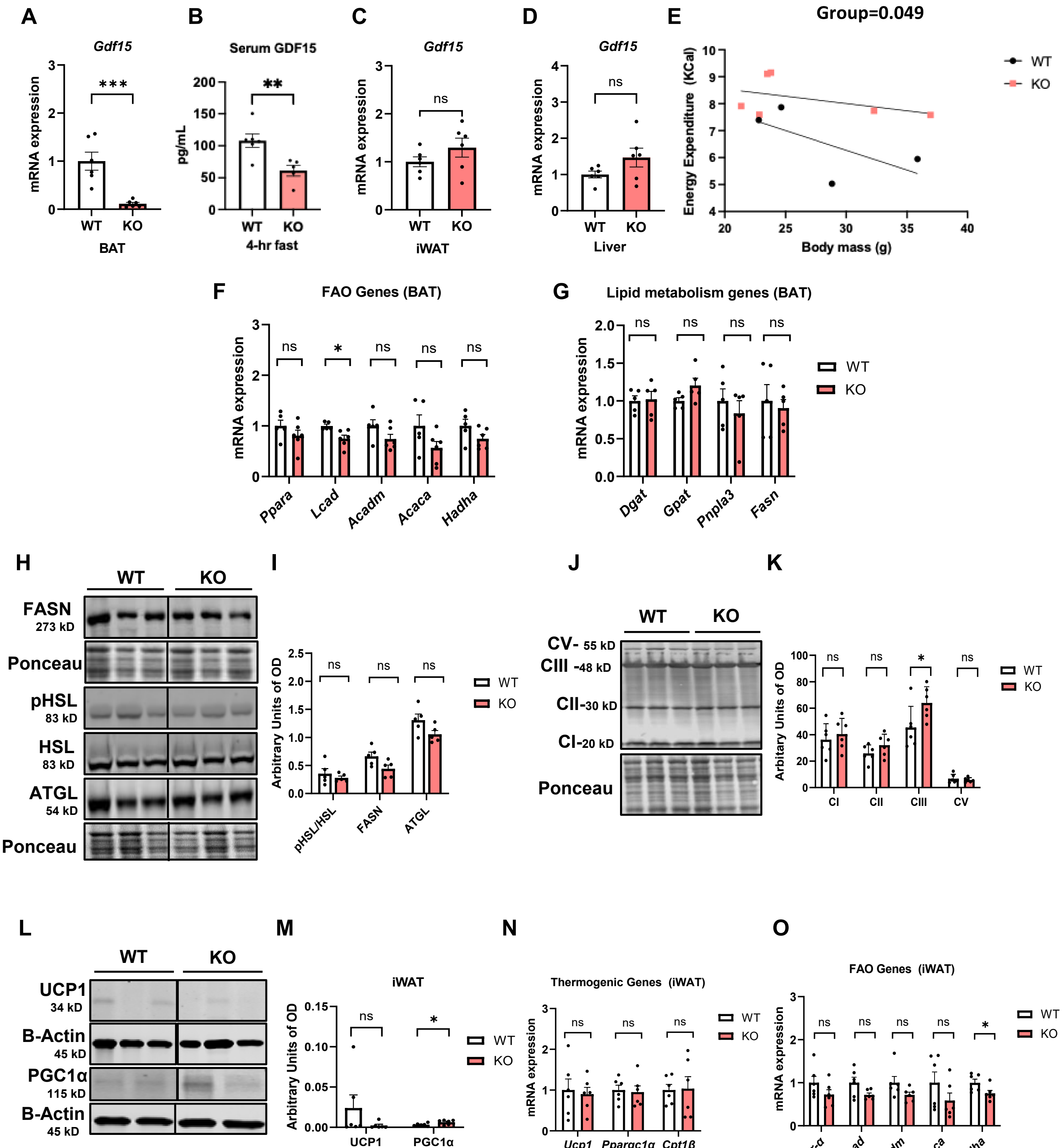

Supplementary Figure 5

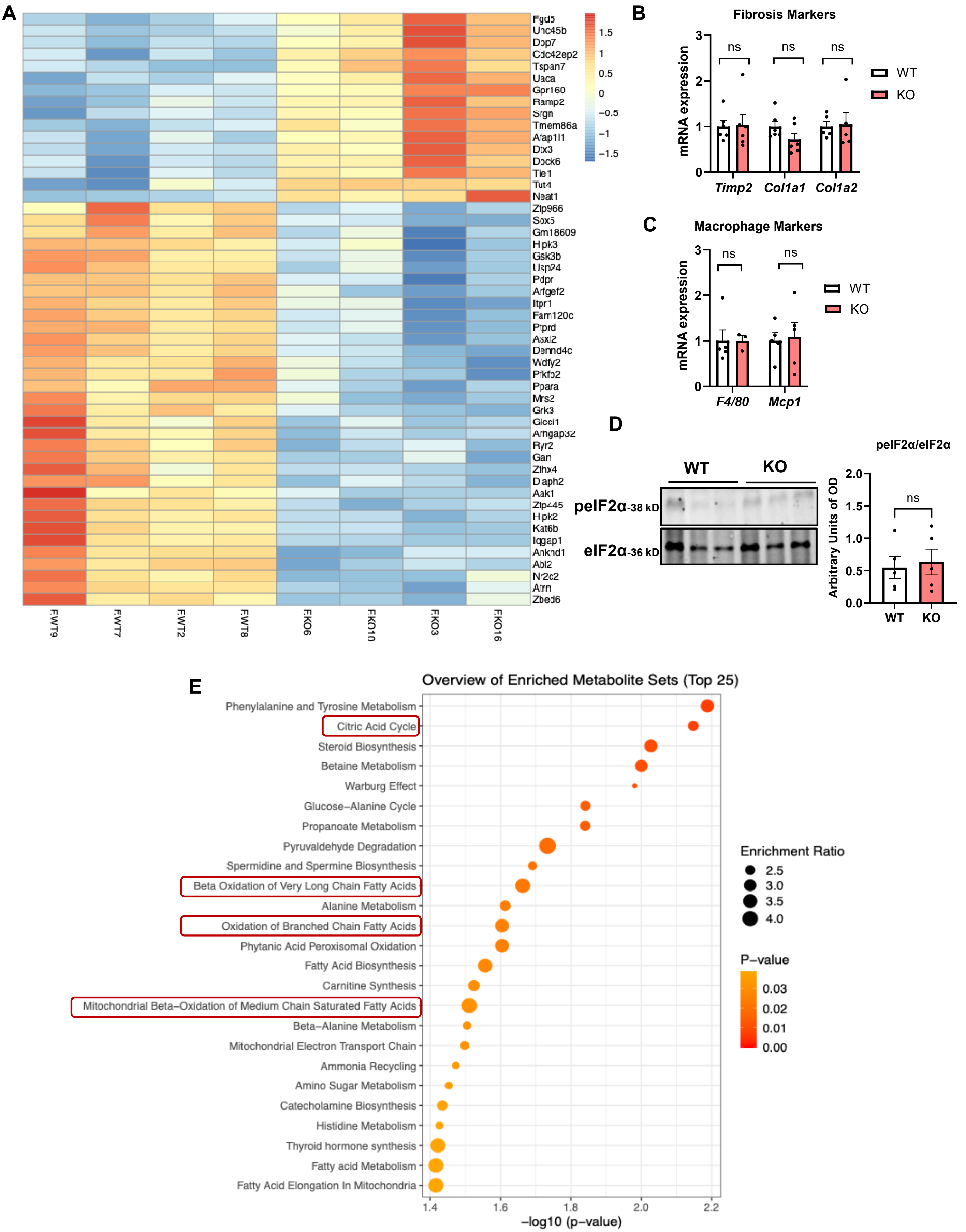

Supplementary Figure 6

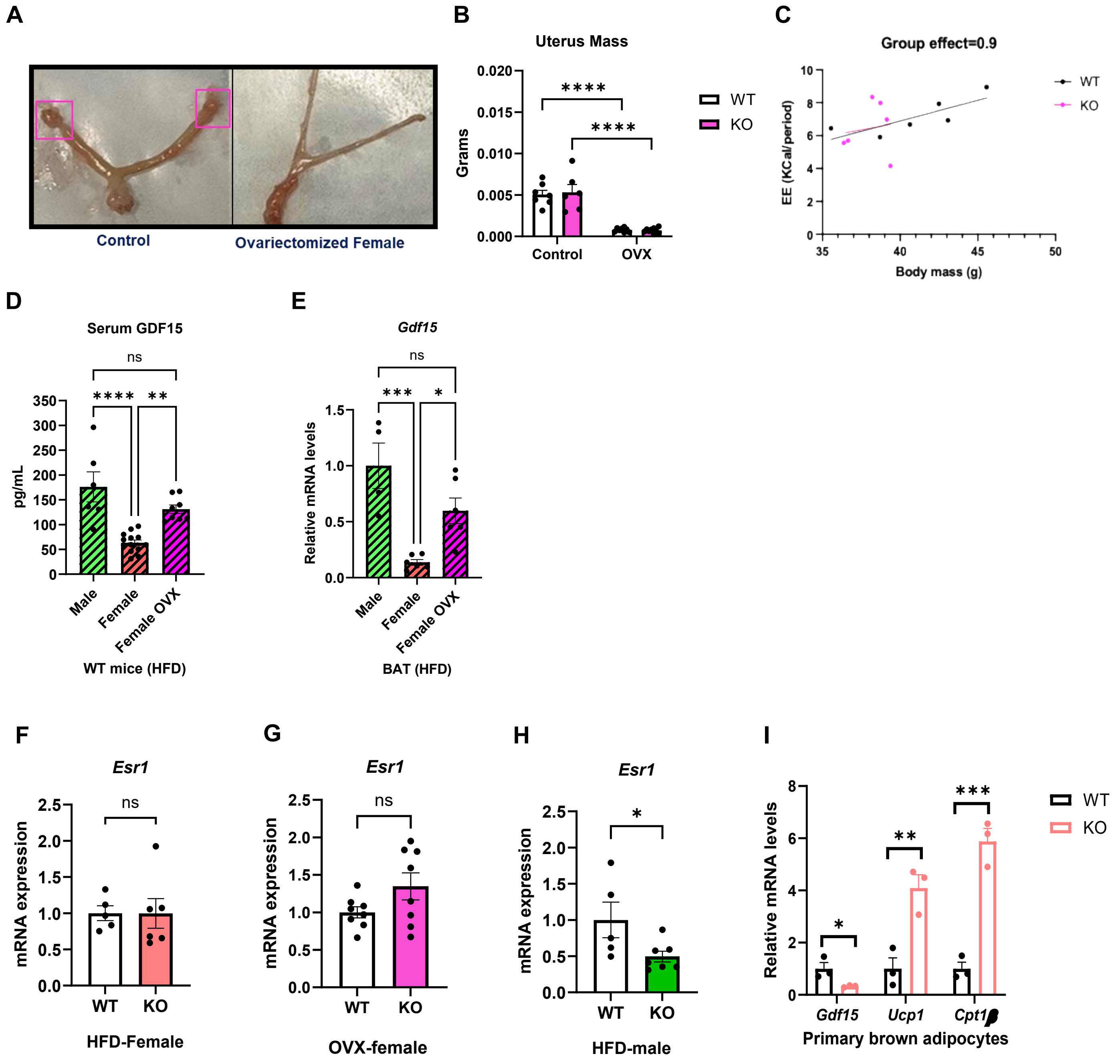

Supplementary Figure 7
